# Overview of Targeted Drugs for Mature B-Cell Non-hodgkin Lymphomas

**DOI:** 10.3389/fonc.2019.00443

**Published:** 2019-06-04

**Authors:** Stefania Crisci, Raffaele Di Francia, Sara Mele, Pasquale Vitale, Giuseppina Ronga, Rosaria De Filippi, Massimiliano Berretta, Paola Rossi, Antonio Pinto

**Affiliations:** ^1^Hematology-Oncology and Stem Cell Transplantation Unit, Istituto Nazionale Tumori, Fondazione “G. Pascale” IRCCS, Naples, Italy; ^2^Department of Clinical Medicine and Surgery, Federico II University, Naples, Italy; ^3^Department of Medical Oncology, CRO National Cancer Institute, Aviano, Italy; ^4^Department of Biology and Biotechnology “L. Spallanzani,” University of Pavia, Pavia, Italy

**Keywords:** anticancer mAbs, tyrosine kinase inhibitors, tailored therapy, personalized medicine, NHL

## Abstract

The improved knowledge of pathogenetic mechanisms underlying lymphomagenesis and the discovery of the critical role of tumor microenvironments have enabled the design of new drugs against cell targets and pathways. The Food and Drug Administration (FDA) has approved several monoclonal antibodies (mAbs) and small molecule inhibitors (SMIs) for targeted therapy in hematology. This review focuses on the efficacy results of the currently available targeted agents and recaps the main ongoing trials in the setting of mature B-Cell non-Hodgkin lymphomas. The objective is to summarize the different classes of novel agents approved for mature B-cell lymphomas, to describe in synoptic tables the results they achieved and, finally, to draw future scenarios as we glimpse through the ongoing clinical trials. Characteristics and therapeutic efficacy are summarized for the currently approved mAbs [i.e., anti-Cluster of differentiation (CD) mAbs, immune checkpoint inhibitors, chimeric antigen receptor (CAR) T-cell therapy, and bispecific antibodies] as well as for SMIs i.e., inhibitors of B-cell receptor signaling, proteasome, mTOR BCL-2 HDAC pathways. The biological disease profiling of B-cell lymphoma subtypes may foster the discovery of innovative drug strategies for improving survival outcome in lymphoid neoplasms, as well as the trade-offs between efficacy and toxicity. The hope for clinical advantages should carefully be coupled with mindful awareness of the potential pitfalls and the occurrence of uneven, sometimes severe, toxicities.

## Introduction

Non-Hodgkin lymphomas (NHL) encompass malignant tumors of the lymphoid tissues variously resulting from the clonal growth of B cells, T cells, natural killer cells, or originators of these cells. They derive from cells at varying stages of maturation, and many of the biologic features of these malignant cells reflect their normal counterparts. B cell lymphomas may arise at any stage of normal B cell development, but most are derived from cells that have been exposed to the germinal center reaction (1). The recent World Health Organization (WHO) classification categorizes B-cell lymphomas by morphology, immunophenotype, and genetic findings. These histological subtypes of B-cell Lymphomas recognized by the WHO present different and somehow specific profiles of clinical aggressiveness and prognosis. Despite, the WHO classification does not explicitly order B-cell lymphomas on the basis of their aggressiveness, also given the significant patient-to-patient variability in the natural history of these neoplasms. Both in real life practice and in the vast majority of clinical trials histological subtypes have been roughly segregated into indolent, aggressive and very aggressive groups, according to their usual clinical behavior. Indolent B-cell lymphomas represent 35 to 40 percent of the non-Hodgkin lymphomas (NHL), and survival is generally measured in years. The most common subtypes include follicular lymphoma (FL), chronic lymphocytic leukemia/small lymphocytic lymphoma (CLL/SLL), a fraction of mantle cell lymphoma (MCL) cases, extramedullary, nodal and splenic marginal zone lymphoma (MZL), and lymphoplasmacytic lymphoma (LPL) (1, 2). Aggressive subtypes if left untreated survive a few months but if adequately treated may achieve definitive remissions and cure in a significant fraction of patients. The most common subtypes are large B-cell lymphomas, including anaplastic and primary mediastinal lymphoma, and various kinds of diffuse large B cell lymphoma (DLBCL). The highly aggressive subtypes represent about 5 percent of the NHL and survival may be measured in only a few weeks if left untreated. Curing is possible if vigorously treated with high-intensity chemotherapy protocols.

Chemotherapy, radiotherapy, and immunotherapy have been used, alone or in combination, in the last decades to treat B-cell NHL. Therapeutic outcomes may vary according to clinical behavior, whether indolent or aggressive, and patients may suffer various patterns of recurrence requiring subsequent lines of rescue therapies. Dismal prognosis still affects a significant fraction of patients with mature B-cell lymphomas, and new treatment strategies should be conceived to improve both objective response and survival (3–9).

In the last decade, the remarkable and exponential understanding of intracellular processes that are deregulated during lymphomagenesis, such as signal transduction pathways, transcriptional and translational regulation, protein stability and degradation, cell cycle regulation, and mitosis and apoptosis, as well as the study of the microenvironment have led to the discovery and progress of new targeted therapies (10–16).

These novel biological therapies include monoclonal antibodies (mAbs), small molecule inhibitors (SMIs) (i.e., growth factors or their receptors), vaccines, and genetic therapies. They may complement or replace conventional chemotherapies (with their burden of systemic toxicities) ensuring novel mechanisms of “targeted” tumor cell kill and proliferation control while, hopefully, lessening iatrogenic adverse events.

Additionally, the role of the immune system in the pathogenesis and development of hematological neoplasms has long been known, but especially in recent years we have seen a significant change in knowledge in this area, such as new open therapeutic perspectives. Using the immunologic mechanism to treat cancer is an old and well-known concept, and it consists in activating the immune system to hit the tumor rather than directly hitting the cancer cell. This approach represents a real change in the treatment paradigm (3, 8, 11, 14, 17–20). Tumor immunotherapy has undergone a new phase of development, in particular linked to the development of T-cell checkpoint inhibitors and the development of CAR T cell therapy, a personalized treatment involving the use of genetically modified T lymphocytes to attack the cancer cells (21–24).

**Figure 1:**
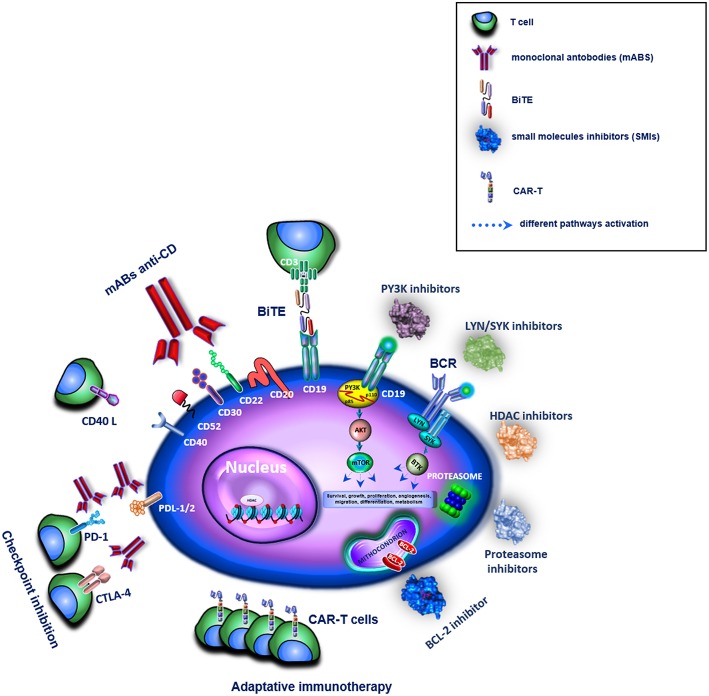
Overview of different target therapies in clinical or pre-clinical use for the treatment of B-cell lymphomas. In the figure these are represented by mABs, BiTE, SMIs, and immune checkpoint inhibitors for an adaptative immunotherapy. The different drugs are shown as family groups based on their different mechanisms of actions.

This review is intended to provide an overview of all Food and Drug Administration (FDA)-approved novel drugs and therapies for “targeting” mature B-cell neoplasms. Immunotherapy agent treatments [i.e., anti-Cluster of differentiation (CD) mAbs, immune checkpoint inhibitors, chimeric antigen receptor (CAR) T-cell therapy, and bispecific antibodies] as well as for SMIs (i.e., inhibitors of B-cell receptor signaling, proteasome and mTOR BCL-2 HDAC pathways) are summarized in their mechanisms of action (Figure 1)—the results they achieved in mature B-cell lymphomas are described in synoptic tables and the ongoing clinical trials are detailed to draw, at a glance, a glimpse on future scenarios.

## Methods

To assess the actual understanding of targeted drugs for NHL, a search on the Cochrane Library and PubMed were performed crossing the keywords “Targeted Therapy” AND “B-Cell Neoplasm.” In the second step “indolent” and “aggressive and very aggressive” were singularly added, limited to the English literature but with no restriction on time. “Monoclonal antibodies” and “Small molecule Inhibitors” restricted the search. The authors examined the titles of the 2090 papers retrieved; 521 of them met the call for monoclonal antibodies while 183 were relevant to SMIs. Most of them were cited in the manuscript.

Papers that did not include anticancer inhibitor series and appeared redundant were excluded. A search for abstracts or full text led to the exclusion of other non-pertinent papers. For studies conducted by the same research institute at different times, the most recent and complete one was included unless different methods, endpoints, or specific issues had been addressed. Papers whose full text or at least abstract were not available were excluded as well. The reference sections of pertinent papers were searched for other relevant articles. Here, we considered novel agents to be the mAbs and SMIs that are in ongoing clinical trials or were in trials that have been completed in the last 2 years.

The Clinicaltrial.gov database was queried regarding the terms of each novel agent and therapy in combination with B-cell lymphoma.

## Monoclonal Antibodies (mAbs)

The therapeutic antibodies targeting cell surface receptors have been employed in the standard care treatments for most cancers, both solid tumors and hematological neoplasms. Therapeutic mABs target specific antigen molecules, such as extracellular growth factors and transmembrane receptors. In some cases, mABs are conjugated with radioisotopes or toxins to allow the specific delivery of these cytotoxic agents to the tumor cell target. In general, the mechanisms that allow therapeutic antibodies to inhibit growth or kill cancer cells fall into two categories: immune-mediated mechanisms as antibody-dependent cell cytotoxicity (ADCC), and complementary cytotoxicity (CDC), and mechanisms that interfere with tumorigenesis pathways (e.g., triggering apoptosis, inhibiting cell proliferation or blocking of angiogenesis) (25).

Herein, for the currently approved mAbs for Lymphomas (Table 1) we recap in four groups the efficacy of (i) anti-Cluster of differentiation (CD) mAbs; (ii) immune checkpoint inhibitors; (iii) chimeric antigen receptor (CAR) T-cell therapy; and (iv) bispecific antibodies.

**Table 1:** Targeted drugs for immunotherapy and signal transduction inhibitors (SMIs) with indications for mature B-cell Lymphomas.

**Drug class**	**Drug (brand name)**	**Target**	**Indication**
**IMMUNOTHERAPY (mAbs and CAR-T)**			
Anti-CD mAbs	Alemtuzumab (Lemtrada)	CD52	CLL/SLL
	Brentuximab vedotin (Adcetris)	CD30	LBCL-ALK+, DLBCL
	Camidanlumab Tesirine (ADC-301)	CD25	DLBCL
	Dacetuzumab	CD40	B-NHL
	Lucatumumab	CD40	CLL/SLL
	Obinutuzumab (Gazyva)	CD20	B-NHL
	Ofatumumab (Arzerra)	CD20	FL B-NHL
	Polatuzumab Vedotin (DCDS4501A)	CD79b	FL, DLBCL, B-NHL
	Rituximab (Mabthera)	CD20	CLL/SLL, LPL,FL, MZL, MCL, DLBCL, HG-BCL
	Ublituximab (TG-1101)	CD20	B-NHL
Immune Checkpoint inhibitors	Atezolizumab (Tecentriq)	PD-L1	FL DLCBL
	Durvalumab	PD-L1	B-NHL, DLBCL
	Ipilimumab (Yervoy)	CTLA-4	B-NHL, FL
	Nivolumab (Opdivo)	PD-1	DLBCL,FL
	Pembrolizumab (Keytruda)	PD-1	DLBCL
	Pidilizumab (MEDI4736)	PD-1	DLBCL
	Urelumab	CD137	CLL/SLL
Chimeric Antigen receptor (CAR) T-Cell Therapy	Axicabtagene ciloleucel	CAR T-4-1BB	DLBCL
	Tsagenlecleucel (CTL019)	CAR T-4-1BB	HG-BCL
Bispecific antibodies	AFM13	CD30/CD16A	DLBCL
	Blinatumomab (Blincyto)	CD19/CD3	DLBCL
	DART	CD19/CD3	DLBCL
	Mosunetuzumab (BTCT4465A)	CD20/CD3	CLL/SLL, iNHL
**SIGNAL TRANSDUCTION PATHWAY INHIBITHORS**			
BCR Inhibitors	Acalabrutinib (Calquence)	BTK	CLL/SLL
	Ibrutinib (Imbruvica)	BTK	CLL/SLL, DLBCL, MCL MZL,
	Buparlisib (BKM120)	PI3K	DLBCL, B-NHL
	Copanlisib (Aliqopa)	PI3K γ	DLBCL, MCL
	Idelalisib (Zydelig)	PI3K δ	CLL/SLL, DLBCL, FL
	Cerdulatinib (PRT062070)	SYK JAK 1-2	FL
	Entospletinib (GS-9973)	SYK	CLL/SLL
	Fostamatinib (Tavalisse)	SYK	DLBCL
	TAK659	SYK/FLT3	DLBCL
Proteasome inhibitors	Bortezomib (Velcade)	PIs	FL, MCL, MZL,
	Carfilzomib (Kyprolis)	PIs	B-NHL
	Ixazomib (Ninlaro)	PIs 20S subunit	NHL DLBCL
mTor inhibitors	Everolimus (Afinitor)		CLL/SLL, DLBCL
	Temsirolimus (Torisel)		DLBCL, MCL
BCL2 Inhibitor	Venetoclax (Venclexta)	BH3 domain	DLBCL CLL/SLL
HDAC Inhibitors	CUDC-907	Class I and II+ PI3K	DLBCL
	Mocetinostat (MGCD0103)	Class I and IV	DLBCL, FL
	Panobinostat (Farydak)	Class I, II and IV	DLBCL
	Vorinostat (Zolinza)	Class I and II	FL

*B-NHL, B-NHL not otherwise specified; CLL/SLL, Chronic Lymphocytic Leukemia/Small Lymphocytic Leukemia; DLBCL, Diffuse Large B cell Lymphoma; FL, Follicular Lymphoma; HG-BCL, High Grade B-cell lymphoma; iNHL, indolent NHL; LBCL, Large B-cell lymphoma; MCL, Mantle Cell Lymphoma; MZL, Marginal Zone Lymphoma*.

### Anti-CD mAbs

In this category are the Anti-CD20 Rituximab and the anti-CD52 Alemtuzumab, the forefathers of the mAbs designed for lymphocyte blocking activities. Both are two chimeric (murine-human) antibodies. The success of rituximab has elicited interest in the development of new agents for other surface antigens on malignant B cells. A new generation of anti-CD20 mABs, including ofatumumab, obinutuzumab, and ublituximab, has been designed with features, distinctive from rituximab, that realize an improvement of ADCC and CDC (26).

**Alemtuzumab** is an anti-CD52 antibody effective in CLL. Currently, it is only accessible on a compassionate use basis (27, 28).

**Brentuximab vedotin** (SGN-35) is a conjugated antibody consisting of a chimeric monoclonal anti-CD30 antibody linked to the strong microtubule inhibitor monomethyl auristatin E (MMAE). After CD30 binding, SGN-35 is internalized, and the MMAE is released by the action of lysosomal enzymes on the valine-citrulline linker. The antineoplastic mechanism of the brentuximab vedotin exerts is still not entirely clear. Dissemination of MMAE in the tumor microenvironment and cytotoxic effects on “spectator cells” may partly explain its action (29). On 2011, it was approved by the FDA for the treatment of Hodgkin lymphoma (HL) patients, but it may also be adopted in cases of ALK-positive large B-cell lymphoma (LBCL) and Primary Effusion LBCL (30–34).

**Camidanlumab Tesirine** (ADCT-301) is a pyrrolobenzodiazepine (PBD) Dimer-containing ADC anti-CD25 (the alpha chain of the IL-2 receptor) (35). CD25 is present on the cell surface in B-cell lymphomas such as DLBCL, further than several T-cell lymphoma subtypes (36, 37).

**Dacetuzumab** is a monoclonal anti-CD40 antibody. A specific gene signature may be predictive of sensitivity to dacetuzumab in patients with DLBCL. It has shown effectiveness as monotherapy in a phase I study of 50 B-cell NHL patients. Almost 33% of patients had a reduction in tumor bulk with an 8 mg/kg/week dose for 4 weeks. In one case a complete response was observed, and five cases showed partial responses (38, 39).

**Lucatumumab** is another monoclonal anti-CD40 antibody. In relapsing CLL, results of the phase I reported that the dosages were well-tolerated in a cohort of 26 patients; 1 patient had a partial response, in 17 cases the disease was stable (40, 41).

**Obinutuzumab** is another humanized anti-CD20-IgG2 class of monoclonal antibody. It retains better ADCC than rituximab, with less CDC than ofatumumab. It has a unique feature in CD20 cross-link, resulting in increased direct cell death. FDA approved obinutuzumab for the treatment of CLL. Also, this mAB has been tested for R/R NHL. A phase III study compared alkylating agent (bendamustine) alone vs. obinutuzumab plus bendamustine followed by maintenance therapy with obinutuzumab in indolent NHL patients refractory to rituximab. The outcomes reported a significantly longer PFS in the of obinutuzumab plus bendamustine arm (24, 42).

**Ofatumumab** is a human mAb direct against a new CD20 epitope. In preclinical models compared with rituximab, ofatumumab has demonstrated a closer linkage with the B-cell surface and enhanced complement-dependent cytotoxicity (43–45).

**Polatuzumab vedotin** is a first-in-class anti-CD79b antibody-drug conjugate (ADC) currently being investigated for the treatment of different NHLs (46, 47). CD79b protein is highly specific and expressed in most of B-cell malignancies (48). To date, some ongoing studies are assessing the safety and effectiveness of polatuzumab vedotin for several types of NHL, including trials exploring combinations with obinutuzumab, rituximab, venetoclax, and atezolizumab (46, 47, 49–51).

**Rituximab** is still the most widely used antibody for treating mature B-cell lymphoma NHL B cells, also including CLL/SLL. Rituximab is an IgG1 chimeric antibody binding to CD20, a B-lymphocyte antigen transmembrane, which is present on the surface of both non-neoplastic (pre, immature, mature, and activated B cells) and malignant B cells (52, 53). The antibody was first approved in 1997 for NHL and subsequently, in 2009, for CLL. After that, rituximab has become an ordinary component of the treatment of FL, DLBCL, and MCL (25).

**Ublituximab** (TG-1101) targets an exclusive epitope on the CD20 and has been engineered to improve affinity for all variants of FcγRIIIa receptors, with better ADCC than ofatumumab and rituximab (54).

#### Commonly Anti-CD mAbs Toxicities

Due to the presence of the entire range of murine immunoglobulins (Igs), mAbs retain a high antigenic potential to humans, therefore carrying a risk for hypersensitivity reactions upon parenteral administration. Indeed, infusional reactions take place quite commonly during or after mAbs administration. Tumor lysis syndrome may occur in patients carrying an elevated number of circulating neoplastic cells. Infusion-related adverse events are equally frequent and may be severe as well, seen also with the new-generation anti-CD20 mAbs ofatumumab and obinutuzumab. The toxicity profile of the Brentuximab vedotin is manageable, though the peripheral neuropathy is an important clinical feature hampering prolonged administration of the drug (29). Patients treated with these new drugs often may form anti-mouse immunoglobulin antibodies, which could counteract the therapeutic effect. To limit these adverse effects, the more recently developed chimeric mAbs contain an increased proportion of human Ig components (about 65%) and a reduced portion of murine Ig components while humanized mAbs account for 95% of the human component (55). Their co-administration with vaccines should be avoided.

### Immune Checkpoint Inhibitors (ICIs)

Immunotherapy has reformed the treatment of solid tumors and hematological neoplasms over the past decade with numerous agents approved by the FDA in recent years. While various approaches are used to modify the antitumor immunity of the host, perhaps the most commonly studied and used is the checkpoint block (15, 56–59). The motivation for adopting ICIs in the treatment of lymphoma relies on the existence in such malignancies of mechanisms that escape immune surveillance due to genetic variance. These agents may re-educate cells in the microenvironment, restoring chemokine and cytokine signaling as well as expression of checkpoint proteins (56, 60–66). They are able to block the cytotoxic T lymphocyte-associated antigen 4 (CTLA-4) and programmed death 1 (PD-1) pathways. PD-1 is an important receptor of the immune checkpoint expressed on activated T cells (67). In recent years, interest in the inhibition of PD-1 in combination with other therapies has increased in the hope of generating a synergistic anti-tumor effect. CTLA-4 is a co-inhibitory receptor expressed primarily in the cytoplasm of inactive naïve T cells. Upon antigen stimulus, CTLA-4 is mobilized to the T cell surface and binds with its ligands CD86 and CD80, causing down-regulation of T cell activation (68–71).

In lymphomas, blocking the checkpoint and harnessing the immune system as antineoplastic therapy is an active area of clinical study. Monoclonal antibodies directed against PD-1 and CTLA-4 are being designed to reduce the down-regulation of T-cell responses against malignant cells (68). Through diminished inhibitory signals, the immune response is improved and able to destroy the malignant cells. The results of the anti-PD-1/PD-L1 block are very exciting in lymphomas with 9p24.1 aberrations such as LBCL primary mediastinal (PMBCL), primary b-Cell testicular and cerebral lymphomas. Less encouraging results are reported for CLL/SLL and most of DLBCL (72). The currently used immune checkpoint inhibitors are the anti-PD-1 mABs Nivolumab, Pembrolizumab, and Pidilizumab, the anti-PDL-1 mAbs Durvalumab, Urelumab, and Atezolizumab, and the anti-CTLA-4 mAb Ipilimumab (73).

The profile of PD-L1 expression by immunochemistry has been lately proposed to retain prognostic and diagnostic significance (24).

**Atezolizumab** (MPDL3280A), is a humanized IgG1 anti PD-L1. It is sustained for use against several hematologic malignancies. Still little is known on the expression of CTLA-4 in human tissue. So far it has been reported that CD80 and CD86, physiological ligands for the expression of CTLA-4, can be observed in T-cell lymphoma patients, in the cells of the dendritic system, and in a subgroup of B-cells of the germinal center and B-immunoblasts in lymphomas (74).

**Durvalumab** (MEDI4736) is a high-affinity human IgG1 mAb that selectively inactivates PD-L1 by binding PD-1 and CD80. It has shown preliminary evidence of antitumor activity across multiple tumor types (68, 75).

**Ipilimumab** is a wholly humanized IgG1 mAb against the CTLA-4. Ipilimumab plus lenalidomide has been reported as well-tolerated after both autologous and allogeneic stem cell transplantation in a phase 2 study achieving a significant proportion of complete responses (76).

**Nivolumab**, a completely humanized IgG4 anti-PD-1 mAB, is now approved for melanoma, non-small cell lung cancer (NSCLC) and renal cell carcinoma. The activity of nivolumab in lymphoid malignancies has also been widely tested (60, 61, 66, 68, 77). Patients with recurrent B-cell NHL were treated at the identical schedule with dose escalation of 1–3 mg/kg of nivolumab. Furthermore, nivolumab as a single agent is undergoing a trial in patients with FL and is currently in phase II studies (NCT02038946). Many ongoing studies also are assessing the effectiveness of nivolumab either in polychemotherapy and/or in combination with other targeted drugs such as ibrutinib (NCT02329847), ipilimumab (NCT01896999), urelumab (NCT02253992), and indoleamine 2,3-dioxygenase 1 (IDO1) inhibitor (NCT02327078). Combinations with ibrutinib or IDO1 are particularly striking in enhancing antitumor T-cell immune responses mechanism (68). Phase 2 trials with nivolumab in patients with DLBCL (CHECKMATE 139, NCT02038933) have mature results. No response was observed in a cohort of MCL patients who receive nivolumab (78).

**Pembrolizumab** (alias lambrolizumab) is a humanized IgG4 antagonistic anti-PD-1 mAb. The usage of IgG4 restricts Fc receptor engagement; this produces the loss of ADCC activity of PD-1- cells, thus enhancing the antitumor immune response. A correlation with a distinctive genetic signature has been described in large B-cell lymphomas also containing alterations and translocations in the number of copies (i.e., 9p24.1/PD-L1/PD-L2) (72). However, several studies on lambrolizumab, either as a single agent (NCT02576990, NCT02362997, NCT02453594, NCT02684292, NCT02535247) and/or in combination with rituximab (NCT02446457), SMIs such as ibrutinib, idelalisib, and IDO1 (NCT02332980, NCT02178722), or conventional chemotherapy (NCT02541565), are ongoing for DLBCL and PMBCL as well as FL and other B cell lymphomas with indolent behavior (56, 74, 79, 80).

**Pidilizumab** was the first humanized IgG1 mAb anti PD-1 to be tested in lymphoid malignancies (56, 68, 74). It is noteworthy that CLL/SLL neoplastic cells show weak PD-1 expression (57), and low numbers of lymphocytes infiltrate PD-1 positive tumors (80). There is evidence of PD-L1 and 2 expressions in a subgroup of NHL, making this pathway a promising target (81).

**Urelumab** is a wholly humanized IgG4 mAb direct against CD137. CD137 (alias 4-1BB or TNFRSF9 receptor) is a member of the growth factor family receptors. CD137 is usually present on the activated T and B cells and monocytes. Although it is not part of the CTLA-4 or PD-1 pathways, its potential to immunostimulatory activities has gained an interest in the clinical development of this mAb. It has been assessed in terms of efficacy and safety in combination with nivolumab and/or rituximab against different subtypes of mature B-cell lymphomas (NCT02253992, NCT02420938) (68).

#### Immune Checkpoint Inhibitors-Toxicities

ICIs are tempting due to their moderately low toxicity profile. The Phase I study in solid tumors reported that 41% of patients treated with nivolumab had an adverse event, and, of them, only 6% were grade 3 or above. The investigators also reported that 71% of patients who received pembrolizumab had adverse events, with 9.5% grade 3 or higher. The main toxicity profile of CTLA-4 and PD-1 inhibitors is associated with its activity in boosting the immune response. Researches on solid tumors report hepatitis, pneumonia, colitis, thyroiditis, hypophysitis, and other inflammatory reactions. Patients receiving therapies with checkpoint inhibitors should regularly be checked for thyroid function and ACTH/cortisol levels if they experience symptoms such as fatigue or hyponatremia (58, 59, 74).

### Chimeric Antigen Receptor (CAR) T-Cell Therapy

It is known that lymphomas are highly susceptible to cellular therapies, including allogeneic stem cell transplantation and the adoptive relocation of specific EBV T cells, which could be seen as the predecessor of the CAR T cells (82). CAR T cells are autologous T lymphocytes genetically modified to bind to specific antigens present on cancer cells. As a result of the binding of CAR T cells to a neoplastic cell, the signaling domains stimulate cytokine secretion, cytolysis of the tumor cell, and T cell proliferation. CAR T cells are created by apheresis of the mononuclear cells from peripheral blood. Successively, the isolated T cells are then transduced *in vitro* with a retroviral or lentiviral vector with a CAR complex including a single-chain variable fragment of antibodies (scFv) or a peptide (21, 22, 24). The later generation (second and third) of CAR cells integrate an additional domain such as CD28 into the construct, which provides a co-stimulator signal. After the expansion of treated T cells, they are ready for infusion into the patient for 1–2 days. Before CAR T cell infusion, patients receive chemotherapy that reduces lymphoma. Ideally, the target antigen of CAR T cells must be absent on healthy cells but present on cancer cells only (24). To date, for hematological malignancies, several CART therapies have received FDA approval. The first was approved was in August 2017 for the treatment of patients aged up to 25 years carrying B-cell precursor acute lymphoblastic leukemia (ALL) to CD19 cell therapy CART-4-1BB (tsagenlecleucel CTL019, Kymriah, Novartis, Basel, Switzerland) (20, 83, 84). In October 2017, the FDA granted regular approval to CD19 CAR T therapy axicabtagene ciloleucel (Yescarta, Kite Pharma, Inc.) for large B-cell lymphoma adult patients relapsed or refractory after two extra lines of conventional therapy. They include high-grade B-cell lymphoma, DLBCL NOS, PMBCL, and DLBCL arising from FL (82, 85–87). However, despite the early efficacy observed in the procedure of CAR-T in the treatment of CLL, the initial trials in other NHLs were less promising than the response rates observed in patients with ALL. With improved induction chemotherapy, which has been demonstrated to trigger the patient for rapid expansion of T cells to adoptive transfer, CAR T cells are now showing a more likely response. There have been two reports from an ongoing study of CAR T cells carrying CD19 receptor composed of a recognition ectodomain ScFv and stimulant endodomain 4-1BB (CTL019) that demonstrate the effectiveness both in DLBCL and FCL (82). In the DLBCL cohort as part of an ongoing phase II study, 40 cases were evaluable for assessing the response at the time of data blocking (NCT03761056).

The lymphodepletion regimen before CAR T cell infusion is dependent on the organization of the institution. Moreover, the protocols for the design of CAR T cells growing and producing lentivirus or retrovirus for cell transduction also differ between studies. The timing of infusion of CAR T cells either after chemotherapy alone or immediately after autologous transplantation need to be standardized. Additional multicenter studies are needed to optimize CAR T cell protocols.

Two CAR-T therapies targeting CD19 on B cell malignancies, Axicabtagene ciloleucel (axi-cel) and tisagenlecleucel, were both effective against multiply recurrent DLBCL. In ZUMA-1, axi-cel resulted in a median duration of response, PFS and OS of 11, 6, and >27 months, respectively (88). In JULIET, relapse-free survival with tisagenlecleucel 1 year after initial response was 65 percent (89). Both agents are associated with serious complications (e.g., fatal neurologic events and cytokine release syndrome), but no new toxicities were identified with longer follow-up. Axi-cel and tisagenlecleucel are approved for use at certified institutions by the US FDA in adults with RR DLBCL after ≥2 lines of systemic therapy.

Several studies report some cases that remain resistant to CAR T cells. The resistance can partly be due to the failure of the CAR T cell to overcome the inhibition created by the neoplastic cells. Therefore, studies are ongoing that combine CAR T cell therapy with inhibitors of the mAB control immune system. One trial being conducted at the University of Pennsylvania is exploring pembrolizumab following CAR T cells (NCT02650999). Another trial at Baylor College of Medicine (Houston, TX, USA) combines ipilimumab with CAR T cells (NCT00586391). An alternative mechanism of CAR T cell deficiency is the absence of perseverance of genetically modified CAR T cells. Research is underway to assess whether cytokine co-administration can improve the clonal expansion of CAR T cells (NCT00968760) (24).

#### CAR T Cells-Toxicities

Cytokine release syndrome (CRS) is possibly one of the leading adverse events of CAR T cell therapy. CRS is related to an elevated number of different cytokines, comprising interleukin-6 (IL-6) and interferon γ. CRS is shown by cumulative adverse events including fever, hypoxia and hypotension. Also, several blood values are altered, such as elevated C-Reactive Protein (CRP), low fibrinogen and highly elevated ferritin. By CAR T cell therapy, the beginning of symptoms correlates with the expansion of T cells, and it is usually evident within days or a few weeks (23). The percentages and severity of CRS therapy in patients with lymphoma are less recurrent than those with high levels of systemic disease such as ALL. The ability of CAR T cells to cross the blood-brain barrier (BBB) and deliver neurological toxicity to the CNS has been documented. A clinical study detected neurological toxicity with CAR T-cell infusion, 3/20 patients presented neurologic toxicity including delirium and 1/20 encephalopathy. The worst of the neurological adverse events are attenuated by the administration of dexamethasone, which also enters the BBB. Due to the exhaustion of non-malignant CD 19 lymphocytes, B cell aplasia is an additional adverse event. Finally, other major adverse events for patients are opportunistic infections due to hypogammaglobulinemia. Hypogammaglobulinemia has efficaciously contrasted with IV immunoglobulin administration after CAR T cell infusion (24, 90–94).

### Bispecific Antibodies

Bispecific antibodies (bs-mAbs) are engineered antibodies able to bind two antibodies in a unique molecule and gain the capacity to target diverse epitopes simultaneously. The Bs-mAbs mechanism is analogous to the CAR-T cells, but unlike the latter, the bs-mAbs are “ready to use” drugs (68, 95–98). The identification of the tumor-specific antigen and straight involving T cells can increase the effectiveness of antibody therapy and minimize the toxicity. A BiTE® (Bispecific T-cell engager) antibody complex consists of a single fusion polypeptide (50–60 kDa) able to link two variable fragments of single chain antibodies (scFv) (99). It carries two specific binding sites, one for a link to specific B cell markers (i.e., CD19) and another that targets a co-stimulator on T cells (i.e., CD3) (24). This simultaneously activity results in T cell activation, proliferation, and T cell-induced target cell lysis (100). Differently to the “living” and self-expandable T cells, bsAbs have a short persistence limit in the patient and low objective in strongly immunosuppressed patients. A fusion of both principles can be the modification of immune or tumor cells to permanently express bispecific molecules (101).

**AFM13** is a bi-specific, tetravalent chimeric antibody construct (TandAb) designed to recruit natural killer (NK) cells via CD16A as immune effector cells to CD30-expressing malignancies. AFM13 will be tried in a larger phase II trial in HL (NCT02321592) in a study with CD30-positive cutaneous lymphoma (NCT03192202) and in combination with pembrolizumab (NCT02665650) (98, 102).

**Blinatumomab** (MT103) is the earliest bispecific construct CD19/CD3 approved by the FDA and the EMA for the cure of R/R ALL. Blinatumomab showed high response rates at very low doses in patients with NHL and ALL B precursor. Blinatumomab contains an anti-CD3 arm and an anti-CD19 arm, allowing the junction of CD3 + T cells with the CD19 + B tumor cells. This mechanism determines the lysis of target cells and resembles T-cell-mediated killing (103, 104).

**DART** proteins (dual affinity retargeting) with a mechanism similar to BiTE® interact with CD3 and CD19. The DART is a new bispecific antibody engineered to overwhelm the mechanical limits of BiTE® to increase stability. DART is composed of diabody-like molecules that have the heavy variable chain (VH) region linked to the variable light (VL) of the second binder, and the VH of the second variable region linked to the VL of the first (96). Early on, DART was revealed to induce cytotoxicity in *in vitro* experiments, exhibited potent activity in several relevant tumors and showed more power than the BiTE® format (105). DART was also revealed to be reliably more effective in eradicating CD19-positive B cells. Notably, without engagement with targeted CD19-positive cells, no activation of T-cells by the DART molecule was observed. Also, *in vivo* in a xenograft mouse model, a DART molecule targeting CD19 assembled using an exclusive anti-T cell receptor antibody portion showed an activity virtually identical to that of the CD19 x CD3 DART molecule (106, 107). The DART setup is mostly attractive for clinical practice since it has been confirmed to have comparable pharmacokinetics with other mAbs. The earliest study on a DART CD19xCD3 was in patients with R/R NHL (96).

**Mosunetuzumab** is a full-length bispecific CD20/CD3 antibody that redirects endogenous T-cells to kill neoplastic B-cells by concomitantly binding to CD3 on T cells and CD20 on B cells. An ongoing multicenter Phase I/IB study (NCT02500407) is evaluating mosunetuzumab in R/R B cell NHL patients. The interim analysis shows that mosunetuzumab monotherapy is clinically active in this cohort of NHL, thus it is showing promising and durable efficacy in FCL and DLBCL.

#### BiTE-Toxicities

Accepting the risk of neurotoxicity and CRS, blinatumomab and other BiTE would be given gradually and with weekly progressive doses (24, 108). A phase I/II study of blinatumomab reported several adverse events including CRS, neurological toxicity (aphasia, ataxia, convulsions, headache, tremor), and leukopenia/neutropenia. Also, in the blinatumomab phase study, < 10% of NHL patients have been grade 3 CRS or higher. Instead, in the phase II study, “early” prophylactic dexamethasone was used for each initiation, and an increased daily blinatumomab infusion dose for 2 days after the start reported no adverse events with CRS (109).

## Small Molecules Inhibitors (SMIs)

Although monoclonal antibodies and other immunotherapies have led to dramatic advances in the treatment of lymphoma patients, the parallel development of small molecule inhibitors has been equally exciting. These SMIs have reformed the therapeutic model for different subtypes of NHL. Many SMIs have been approved by the FDA, and others are still under evaluation. Several SMIs are administered orally, are moderately well-tolerated and offer patients unprecedented response rates. Their small size (**≤**500 Daltons) allows for interchange through the plasma membrane, enabling the interaction with intracellular signaling molecules and the cytoplasmic domain of cell surface receptors. These SMIs inhibit signal transduction pathways by targeting proteins involved in transcriptional/translational regulation, protein stability, cell cycle regulation of mitosis and apoptosis. These new agents are a heterogeneous group of drugs with different mechanisms of action: (i) B cell receptor signaling Inhibitors like TKI, BKI, Aurora Kinase Inhibitors (AKI), and SYK; (ii) proteasome inhibitors and; (iii) HDAC inhibitors (Table 1). The SMIs are not free from toxicity, especially when combined with other drugs. Therefore, we will provide advice on the relevant toxicity profiles, because these promising new treatments could hide pitfalls for the treatment of patients with NHL. In clinical practice, these new agents generate a multifaceted step in pharmacokinetics (PK), which does not encompass broad individual PK variability and unpredictable outcomes according to the pharmacogenetic profile of the patient (e.g., cytochrome P450 enzyme) (10, 17, 110, 111).

### B Cell Receptor Signaling Inhibitors

Signaling mediated by B cell receptors (BCR) plays a fundamental role in the expansion of B cell neoplasms. Antigenic stimulation of the BCR extracellular domain starts a signaling cascade accountable for several B cell functions and proliferation. This signal leads to the enrollment of CD79a and CD79b, leading to activation of the spleen tyrosine kinase (SYK) and the LYN kinase. SYK and LYN phosphorylated tyrosine-based immunoreceptor activators activate Bruton tyrosine kinase (BTK) and inositol phosphatidyl three kinase δ (PI3Kδ) (111–115). Inhibitors of BTK, PI3Kδ, and the SYK have been designed to block kinases in this way (17).

#### BTK Inhibitors

**Ibrutinib** (PCI-32765) is an irreversible oral inhibitor of BTK that binds the active site cysteine-481 (Cys481) of the BTK enzyme. BTK is mainly expressed on—but not limited to—B cells, and ITK is mainly expressed on T cells (111–113). Though chemoimmunotherapy is the standard of care for patients eligible with CLL, its toxicity and risk of infection exclude its use in frail patients (elderly and those with co-morbidities). Another restriction to the treatment group consists of patients carrying 17p aberrations of the TP53 gene as poorly endowed with ordinary chemoimmunotherapy (116, 117). The combination of ibrutinib with mAbs also led to high response rates, with ORRs of 95% with rituximab, 71–100% with oratituumab, and 88% with ublituximab (118, 119). However, there was abrogation of induced lymphocytosis from therapy although it is not yet clear how meaningfully the combination affects the deepness and duration of the response (DOR) equated only to ibrutinib (17). Ibrutinib has been tried in combination with rituximab, ifosfamide, carboplatin, and etoposide (R-ICE). It is also used, as well as rituximab, gemcitabine, dexamethasone, and cisplatin (R-GDP), in the second line rescue therapy for R/R DLBCL patients. Ibrutinib was evaluated in R/R FCL and R/R MCL in several clinical trials as monotherapy and combinations.

The second generation BTK inhibitors include acalabrutinib (ACP-196) and underdevelopment ONO-4059 (GS-4059), BGB-311, and CC-292. **Acalabrutinib** is an irreversible BTK inhibitor with a shorter pharmacokinetics t_1/2_. It does not inhibit EGFR and other TK receptors. In a phase I study, 95% of patients with R/R CLL carrying the 17p alteration accounted for the median at a follow-up of 14 months. A common adverse event was diarrhea and bleeding, but no atrial fibrillation was reported (120). It is improbable that ACP-196 is effective in patients with ibrutinib resistance (120). But its use in intolerance to ibrutinib patients is now under investigation (NCT02717611). Other second-generation BTK inhibitors have accounted for effectiveness (121–123). It remains to be understood whether these molecules will have a noteworthy effect compared to ibrutinib.

### BTK-Toxicities

The most common adverse effects were non-hematologic toxicities, including muscle spasms, nausea, fatigue, diarrhea, skin rash, and arthralgia. Hematologic toxicities were less common and included several grades of neutropenia, thrombocytopenia, and anemia (124).

#### PI3K Inhibitors

More downstream from BTK is PI3K. Ubiquitous PI3K fits a highly conserved family of kinases with specific tissue isoforms α, β,γ, and δ. The isoform δ is present on leukocytes and is, therefore, a target of interest. The γ isoform has been associated with the growth and signaling of T cells. The inhibition of p110δ has been revealed to reduce the downstream signaling of the BCR, CXC 4 receptor (CXCR4), and 5 (CXCR5) chemokines. In preclinical studies, it resulted in decreased protein kinase B (AKT) activation, a molecular target of rapamycin (mTOR) and other pathways (111). The PI3K inhibitors currently in use and under investigation in lymphomas are Idelalisib, Copanlisib, Buparlisib, and Umbralisib. Overall, PI3K inhibitors seem to have low response rates in patients with R/R DLBCL when used as monotherapy. It should be studied in combination with other new agents with carefulness to minimize latent toxicity (125).

**Buparlisib** is a strong PI3K oral inhibitor that has confirmed effects in *in vitro* and *in vivo* models of hematologic malignancies (126–128).

**Copanlisib** is an intravenous class I directed against isoforms PI3K-γ and PI3Kδ (129). To assess the effectiveness of copanlisib in DLBCL, patients were treated with 60 mg (130). Copanlisib was evaluated in both indolent and aggressive lymphomas (130–133).

**Idelalisib** (CAL-101) is a potent and highly specific inhibitor of the PI3K δ isoform. It is approved for refractory indolent lymphoma (134, 135). Idelalisib has shown activity either as a single agent and/or in combination with mAbs in R/R CLL in FCL and HL (136–142).

**Duvalisib** is an oral inhibitor of PI3K δ and γ isoforms showing activity in the small non-randomized study of patients with multiply relapsed FL. It is approved by the FDA as a single agent for the treatment of relapsed FL patients who received at least two previous conventional therapies. In this study, CRs are quite uncommon although ~40% of patients achieve a PR. More recently, a small single-arm multicenter trial (DYNAMO) of duvelisib in multi-relapsed patients with CLL/SLL, MZL, and FL reported response rates over 40 percent with an estimated median duration of response of 9.5 months. CLL/SLL patients had a better outcome than the other subtypes (143). Fatal and/or serious toxicities could be seen, including opportunistic pneumonitis from *P. jirovecii* pneumonia, diarrhea or colitis, and cutaneous reactions.

**Umbralisib** is the latest oral inhibitor of both PI3Kγ and casein kinase 1ε (CK1ε).

#### PI3K Inhibitor Toxicities

PI3K inhibitors have a distinctive toxicity profile, including severe diarrhea/colitis. Grade 3 or higher toxicity has been reported with an incidence of around 15%. In addition, opportunistic infections including pneumocystis jirovecii pneumonia (PJP) and cytomegalovirus (CMV) have been recognized in patients treated with idelalisib (144–146).

### SYK Inhibitors

Other components of BCR signaling are potential targets include LYN and SYK as described above (20). SYK is an SH2 domain-containing tyrosine kinase activity. Constitutive activation by SYK leads the development of NHL. It is noted that DLBCL tissue overexpresses the components of the BCR signaling pathway, including SYK. Inhibition of SYK remains a promising goal, but it should be combined with other drugs to produce lasting and meaningful responses (147).

**Cerdulatinib** (PRT062070) is an oral kinase dual inhibitor of JAK 1/3 and SYK and has been revealed in *in vitro* experiments to have a specific inhibitory action in a subgroup of B-cell lymphoma cell lines (148). Cerdulatinib inhibited B-cell activation in a murine model of chronic BCR stimulus. In DLBCL cell lines, cerdulatinib induced apoptosis, blocking cell-cycle, BCR and JAK/STAT signaling (149). It has been described as having synergistic action of cerdulatinib and venetoclax in primary a CLL primary cell line (150). Remarkably, cerdulatinib showed better inhibition of cell duplication than ibrutinib in the ibrutinib-resistant CLL cells and BTKC481S-transfected/ibrutinib-resistant lymphoma cells (147, 151, 152). This double SYK/JAK inhibitor was also evaluated in patients with different R/R B Cell malignancies (153, 154).

**Entospletinib** (GS-9973) is an oral drug that selectively inhibits SYK (155). This 2nd generation molecule showed increased *in vitro* and *in vivo* selectivity for JAK-2, c-KIT, FMS-like tyrosine kinase 3 (FLT 3), VEGFR2, and RET compared to fostamatinib (155). In a multicenter study on subjects with R/R CLL and NHL, entelospletinib showed a promising toxicity profile (147). Moreover, in the latest phase II study entospletinib was shown to have low clinical activity in 39 patients with R/R MCL (147, 156).

**Fostamatinib** is an oral Syk inhibitor leading to a reduction in cell survival (157). In this light, good preliminary results were obtained from a double-blind, randomized study enrolling patients with R/R DLBCL who were not suitable for HSCT (158–160).

**TAK659** is a promising selective, reversible SYK and FLT3 inhibitor demonstrated in both *in vitro* and *in vivo* models (161). Inhibition of SYK remains a promising goal, but it should probably be joined with other antineoplastic drugs to harvest lasting and significant responses (111).

#### SYK Inhibitors Toxicities

The most frequent toxicities observed with SYK Inhibitors are diarrhea, nausea, hypertension and fatigue. Hematological common adverse events are neutropenia and thrombocytopenia (147).

### Proteasome Inhibitors (PIs)

The ubiquitin-proteasome pathway is a multifaceted complex responsible for the regulation of proteins involved in neoplastic activity, such as cyclin-dependent kinases (CDK), BCL-2, and NFκB complex (162, 163). The role of the proteasome is upregulation of these key pathways, making it a promising antineoplastic target (10, 164–168). Finding that PIs lead to cell cycle inhibition and apoptosis in tumor cells has pushed them to be developed as antineoplastic agents. The studies revealed a complex system of ubiquitin ligases and related proteins that orchestrate the delicate balance of longevity of proteins within cancer cells. It is thought that the constellation of proteins whose degradation is inhibited by PI interrupts intracellular processes crucial for the survival of tumor cells. Some examples are (1) cell cycle interruption by inhibiting the degradation of CDK such as p21 and p27; (2) inhibition of the nuclear signal transduction pathway of the κB factor (which typically inhibits apoptosis) through the accumulation of the I-κB inhibitory protein; and (3) promoting apoptosis prolonging the function of the pro-apoptotic members of the Bcl-2 proteins, such as Noxa (10, 164–168).

**Bortezomib** was the first of this class of drugs to undergo clinical development. The first phase 1 study of hematological malignancies showed signs of activity in multiple myeloma (MM), FCL, MCL, and MALT lymphoma (169, 170). It is FDA approved for use in naive and R/R multiple myeloma (MM).

Bortezomib's success has triggered the evolution of 2nd generation PIs, looking to improve on the activity but to minimize the toxicities (primarily peripheral neuropathy) not only for MM but also as therapeutic alternatives in other diseases, including lymphomas and systemic amyloidosis. The development of **carfilzomib** has shown noteworthy advancement to being effective and less neurotoxic for patients with relapsed or R/R MM who failed ≥1 preceding line of therapy. Unlike carfilzomib, bortezomib has demonstrated irreversible inhibitory kinetics.

**Ixazomib** is a second-generation inhibitor of the 20S proteasome that is supplied in both IV and oral drug formulations. Ixazomib has shown efficacy in preclinical lymphoma models (171, 172). This PI has a modest single-agent activity, although so far combination with other drugs has not been shown to increase overall results (111, 173).

#### Proteasome Inhibitors Toxicities

Proteasome Inhibitors showed a significant toxicity profile: serious neurotoxic side effects, cardiovascular and gastrointestinal toxicities, peripheral neuropathy and cytopenias (174–176). Other symptoms include herpetic zoster reactivation lymphopenia, thrombocytopenia, and persistent fatigue. In addition, although rare a minor proportion of subjects showing cardiac failure was recorded with the 1st generation of PIs (177).

### Mammalian Target of Rapamycin-mTOR Inhibitors (mTOR)

mTOR is a keyway in the regulation of trans-membrane trafficking, protein degradation, ribosome biogenesis, protein kinase C signaling, and DNA transcription (178, 179). Thus, triggering of the PI3K/AKT pathway and mTOR signaling is essential in lymphomagenesis. Inhibition of this pathway revealed the blocking of cell duplication (180–182).

**Everolimus** is an oral mTOR inhibitor (183). The primary studies examined this agent in R/R DLBCL, and afterward for R/R CLL/SLL and R/R HL (184–190).

**Temsirolimus** is an FDA-approved IV mTOR inhibitor in metastatic renal cell carcinoma (191). The EMA in Europe approved Temsirolimus for MCL, too (192), and temsirolimus was also added to the list of rescue regimens for R/R NHL patients (193, 194).

#### mTOR Inhibitor Toxicities

mTOR inhibitors are attractive agents since they are well-tolerated as single agents and in combination with other drugs. They have also demonstrated synergism with PIs, leading to the study of combination therapy (10). The side effects include a variety of metabolic, hematological, respiratory, renal, and dermatological toxicities. The tolerability scale of mTORIs, even at the same dosage and for the same application, ranges from excellent to debilitating (e.g., buccal aphthous), can sometimes be fatal (pneumonitis) and may occur at different time points (from days to years) after the initiation of rapalog therapy. Surprisingly, the rate of some side effects, such as pneumonitis or mucocutaneous effects, seems to increase with the dosage of the drug, whereas mTOR is inhibited at the nanomolar range by rapalogs. Alternatively, the majority of these side effects are idiosyncratic and unpredictable (195–197).

### BCL2 Inhibitor

Several neoplasms seem to be mainly dependent on a specific balance of Bcl-2 family expression for their survival, and Bcl-2 overexpression can lead to both *de novo* and acquired chemoresistance (10). The overexpression of the anti-apoptotic BCL-2 protein is frequent in several NHL subtypes, including 30% of the DLBCL (198–201). Inhibition of BCL-2 has become an important treatment strategy because of increasing apoptosis. BCL2 inhibitors were applied primarily for the treatment of CLL patients (111, 202, 203).

**Venetoclax** is an oral formulation. In preclinical study, it has been shown to have powerful selective “BH3-mimetic” activity independent of BCR signaling (apoptosis free of p53) (204–207). In xenotransplantation models, venetoclax has shown greater efficacy when combined with chemoimmunotherapy. Despite the recurrent overexpression of BCL2, monocomponent venetoclax did not have an equally robust response as expected in the DLBCL, while it seems to be well-tolerated (206, 208). Forthcoming studies focus on multiple combinations of venetoclax to increase responses. The European Commission of Medicines (EMA) has approved the combination of venetoclax plus rituximab (V + R) for the treatment of R/R CLL patients previously treated by other therapies (209–211). EMA approval is established on the published results of the Phase 3 MURANO randomized trial (210, 212, 213). This trial compared the BCL2 inhibitor venetoclax administered up to a maximum of 2 years, associated in the first 6 months of R treatment, with the classic chemo-immunotherapy regimen bendamustine and rituximab (BR) administered for six cycles every 4 months (210, 213).

#### BCL2 Inhibitor Toxicities

Nausea, diarrhea, anemia, lymphopenia, neutropenia, and thrombocytopenia are the most frequent AEs, with a minor—although enough to grab attention—incidence of tumor-lysis syndrome (214–216).

### HDAC Inhibitors (HDIs)

Histone deacetylases (HDAC) are enzymes designed against both the histone and non-histone proteins. To date, 18 HDAC enzymes were identified based on their homology with yeast deacetylases. Human HDACs were categorized into four classes: class I includes HDAC 1, 2, 3, and 8, which are located in the nucleus. Class II comprises HDAC 4, 5, 6, 7, 9, and 10, which have a mutable cellular location; class III contains the NAD-dependent yeast homologs, SIRT 1-7, which are not targeted by the currently available HDAC inhibitors (HDACI). Finally, class IV includes HDAC 11 (217, 218). HDIs have been shown to activate cell cycle checkpoints, promote apoptosis, induce cell differentiation, suppress angiogenesis, and improve immune surveillance. HDAC inhibitors (HDI) include a class of synthetic or natural chemical compounds that inhibit the enzymatic activity of HDAC. Several HDIs have been studied in lymphomas, demonstrating only modest clinical benefit, and other HDIs are currently studied in preclinical studies (218, 219).

**CUDC-907** is a class I-II oral double-inhibitor of HDAC and PI3K (α, β, γ) enzymes (111, 220, 220–222).

**Mocetinostat** is an oral HDI that inhibits class I and IV, specifically HDAC isoforms 1, 2, 3, and 11 (223, 224). Mocetinostat was evaluated in a phase II study in R/R DLBCL patients (18, 111, 223–225).

**Panobinostat** is a potent pan-HDAC inhibitor with low dosage achievement against class I, II, and IV HDAC and is FDA approved for DLBCL (225).

**Vorinostat** is one of the first HDAC inhibitors with activity against HDAC class I and II. It has synergistic antineoplastic action when combined with topoisomerase II inhibitors (111, 226–228).

#### HDAC-toxicities

Even though the HDAC family contains several chemical compounds with selectivity for different HDAC isoforms, they unexpectedly have analogous toxicity profiles. Generally, common non-hematologic AEs are diarrhea, nausea, vomiting, fatigue, anorexia, weight loss, and asthenia. Most common hematologic AEs are thrombocytopenia, anemia, and neutropenia (229).

## Novel Agents in Mature B-Cell Lymphoma Subtypes With Indolent Behavior

Patients suffering mature B-cell lymphoma with histological subtypes associated with an indolent behavior such as CLL/SLL, FL, MZL, LPL, and a fraction of those with MCL are generally highly responsive to chemotherapy regimens conventionally based on purine analogs, alkylators with or without the inclusion of anthracyclines. However, they remain still incurable and suffer subsequent relapses and a high risk of histological transformation toward a “large cell” histology. Targeted agents have redefined treatment paradigms in this setting of recurrent patients (Table 2). Most patients with FL experience serial relapse and will be treated with many available agents at some point during their disease course. A preferred order for their use has not been established. Novel agents such as idelalisib, copanlinib, or duvelisib and radioimmunotherapy may be used for multiply relapsed indolent B cell lymphomas. The efficacy and safety of novel agents may quietly differ among different subtypes. As an example, Ibrutinib, which achieves high response rates in MCL, accounts for only 21 and 38 percent ORRs in patients with R/R FL, respectively. Results in the setting of recurrent patients have prompted some of these agents, targeting either cell surface antigens, intracellular pathways or the microenvironment, as a possible front-line option (**Table 4**).

**Table 2:** Overview of the efficacy of select novel therapies in Mature B-Cell neoplasms: indolent histology.

	**Authors**	**Drug**	**Target**	**Phase**	**Setting**	**N^**°**^ of pts**	**ORR% (CR %)**	**PFS% (y)**	**PFS median (mo.s)**	^*^AEs
CLL/SLL	Byrd et al. (121)	Acalabrutinib	BTK	I	R/R with 17p alteration	61	95%	NR	14.3	Dyarrhea
CLL/SLL	Byrd et al. (121)	Ublintuximab	CD20	II	Naïve. Two arms of 1 g/day and 2 g/day	80	67%	NR	20.3	12% neutropenia in 2 g/day arm
CLL/SLL	Nastoupil et al. (230)	Ublintuximab	CD20	I	Dose escalation. Post-Rituximab. In combination with umbralisib, and ibrutinib	46	84%	NR	NR	24%
CLL/SLL/	Ding et al. (83)	Pembrolizumab	PD-1	II	R/R carrying 17p alteration and IGHV unmutated	16	44%	NR	NR	ND
CLL/SLL	Gopal et al. (139)	Idelalisib	PI3K	II	R/R. In combination to Brivatinib	125	57% (6%)	12% (2 y)	11	ND
CLL/SLL	Liu et al. (147)	Entospletinib	Syk	II	R/R. dosing 1,6 g/daily	41	61%	NR	13.8	ND
CLL/SLL	Seymour et al. (210)	Venetoclax	BCL2	II	R/R. 17p deletion In combination to Rituximab	49	86%	82% (2 y)	NR	67%
CLL/SLL	Jaglosky et al. (119)	Ibrutinib	BTK	1b/II	R/R with 17p deletion. Dosing 420mg/day. In combination to Ofatumumab	71	83% (1.5%)	83% (1 y)	NR	11% led discontinuation
CLL/SLL	Rosenthal et al. (18)	Ibrutinib	BTK	III	R/R. In combination to Bendamustine and Rituximab	ND	93% 40%	96% (1 y)	NR	ND
FL	Younes et al. (231)	Nivolumab	PD1	I	R/R in combination to ibrutinib	40	36%	NR	5	13% anemia
FL	Ganjo et al. (232)	Ocaratuzumab	CD20 FcγRIIIa	I/II	R/R with low affinity genotype FcγRIIIa	50	30%	NR	9.2	ND
FL	Czuczuman et al. (233)	Ofatumumab	CD20	I/II	R/R. Dosing 500 mg/day	27	22%	NR	5.8	Neutropenia
FL	Westin et al. (234)	Pidilizumab	PD1	II.	R/R. Dosing 3 mg/kg IV every 4 weeks. In combination to Rituximab	32	52%	NR	15.4	No AEs grade >2
FL	Westin et al. (234)	Pidilizumab	PD-1	II	R/R combined with Rituximab	29	66%	NR	18.8	No AEs grade >2
FL	Gopal et al. (141)	Idelalisib	PI3Kd	II	R/R. Dosing 150mg twice daily	125	57% (50%)	NR	11	Neutropenia 27%
FL	Bartlett et al. (235)	Ibrutinib	BTK	I	Naïve in combination with Rituximab. Dosing 560mg/day	31	37.5% 12.5%	80.4% (2y)	14	Neutropenia 10%
FL	Davids et al. (236)	Venetoclax	BCL2	I	R/R to Bendamustine Rituximab. Dosing 1.2 g/day	29	38%	NR	11	Neutropenia 11%
MZL	Noy et al. (237)	Ibrutinib	BTK	II	R/R. dosage 560 mg/day	63	48%	NR	14.2	pneumonia 8%
B-NHLnos	Goebler et al. (109)	Blinatumumab	CD3/CD19	I	R/R maximum dose tolerated	35	69%	NR	13,5	ND
B-NHLnos	Ansell et al. (238)	Ipilimumab	CTLA-4	I	R/R. 3 mg/kg/mo.s × 4mo.s	18	11%	NR	16	ND
iNHL	Cheson et al. (45)	Obintuzumab	CD20	III	Randomly comparing to bendamustine in R/R to rituximab	396	NR	NR	22.5	ND

* *Grade ≥3 non hematological AEs, only*.

*ASCT, Autologous Stem Cell Transplantation; B-NHL, B-NHL not otherwise specified; CLL/SLL, Chronic Lymphocytic Leukemia/Small Lymphocytic Leukemia; DLBCL, Diffuse Large B cell Lymphoma; FL, Follicular Lymphoma; HG-BCL, High Grade B-cell lymphoma; IGHV, Immunoglobulin G Heavy Variable chain; iNHL, indolent NHL; IV, intravenous; LBCL Large B-cell lymphoma; MCL, Mantle Cell Lymphoma; mo.s, months; MZL, Marginal Zone Lymphoma;; ND, Not Documented NR, Not Reached; pts, patients; R/R, Refractory/relapsed; y, years*.

## Novel Agents in Mature B-Cell Lymphoma Subtypes With Aggressive and Very Aggressive Behavior

Due to the high failure rate produced by excessive toxicity and low response rates to conventional chemotherapies (or both), subtypes with aggressive behavior such as DLBCL, the majority of MCL, transformed FCL and Burkitt lymphoma still represent a burning problem and an unmet need in the setting of mature-B cell lymphoma.

The myriad of novel agents under development, targeting the new pathways fundamental to aggressive B cell growth is expected to offer added clinical benefit to patients with aggressive B cell NHL. Furthermore, these novel agents characterize sustained advancement in the planning for individualized therapies, as single modality treatment, or combined with chemotherapy or other targeted agents (Table 3).

**Table 3:** Novel agents currently under investigation in Mature B-Cell neoplasms: aggressive and very aggressive histology.

**Subtype**	**Author**	**Drug**	**Class/target**	**Phase**	**Setting**	**N^**°**^ of pts**	**ORR%**	**PFS % (y)**	**PFS (mo.s)**	^*^AEs
DLBCL	Ansell et al. (63)	Nivolumab	Anti-PD1	II	R/R Failed to ASCT (87 pts). Ineligible to ASCT (34 pts)	121	10% failed 3% ineligible	NR	Failed 12.2 Ineligible 5.8	24%
DLBCL	Armand et al. (239)	Pidilizumab	Anti-PD1	II	R/R ASCT	66	51%	NR	16	ND
DLBCL	Locke et al. (88)	axicabtagene ciloleucel	CD19	I/II	R/R. 1.0 × 10^6^ CAR T cells/Kg	101	83% (58%)	NR	5.9	11%
DLBCL	Shuster et al. (89)	tisagenlecleucel	CD19	II	R/R. 1.0 × 10^7^-6.0 × 10^8^ CAR T cells	93	52% 40%	35% (1 y)	NR	ND
DLBCL	Viardot et al. (110)	Blinatumumab	CD3-CD19	II	escalation dose 9-28-112 ug/day	17	43%	NR	NR	17% Neurologic
DLBCL	Wang et al. (240)	Ibrutinib	BTK	II	R/R	54	28%	NR	3	ND
DLBCL	Younes et al. (241)	Buparlisib	PIK3	II	R/R	26	11.5%	NR	1.8	Hyperglicemia 11%
DLBCL	Flinn et al. (158)	Fostamatinib	Syk	I/II	Ineligible for ASCT. Dosing 200 mg/day	47	21% (4%)	NR	5.3	ND
DLBCL B-NHL	Rhodes et al. (112)	TAK659	Syk/FLT3	II	R/R	77	27%	NR	NR	ND
DLBCL	Witzens-Harig et al. (194)	Temsirolimus	mTOR	II	In combination with rituximab. Dosing 24, 50, 75, or 100 mg	32	28% (12.5%)	NR	2.6	ND
DLBCL	Rhodes et al. (112)	Vorinostat	HDAC	I/II	In combination to R-CVEP	16	57%	NR	9.2	ND
DLBCL FL	Batlevi et al. (225)	Mocetinostat	HDAC	II	R/R	72	18.9%	NR	2.1	ND
DLBCL	Oki et al. (242)	CUDC-907	HDAC/PIK3	II	R/R (14 of them with Myc altered). with or without Rituximab	37	37% 64% in Myc altered	NR	11.2 13.6 in Myc altered	Neutropenia
HG-BCL	Dryling et al. (131)	Copanlisib	PI3K-γ and PI3Kδ	II	CD79b mutations	43	25%	NR	2 m	ND
MCL	Wang et al. (243)	Lenalidomide	PIs	II	R/R to Ibrutinib	58	29%	NR	5	No AEs grade >2
MCL	Younes et al. (241)	Buparlisib	PIK3	II	R/R	22	22.7%	NR	11.3	ND
MCL	Jerkeman et al. (244)	Ibrutinib	BTK	II	R/R in combination to Rituximab and Lenalidomide	50	76%	NR	17.8	Neutropenia 38%, Infection 22%

* *Grade ≥3 non-hematological AEs, only*.

*ASCT, Autologous Stem Cell Transplantation; B-NHL, B-NHL not otherwise specified; CLL/SLL, Chronic Lymphocytic Leukemia/Small Lymphocytic Leukemia; DLBCL, Diffuse Large B cell Lymphoma; FL, Follicular Lymphoma; HG-BCL, High Grade B-cell lymphoma; IGHV, Immunoglobulin G Heavy Variable chain; iNHL, indolent NHL; IV, intravenous; LBCL Large B-cell lymphoma; MCL, Mantle Cell Lymphoma; mo.s, months; MZL, Marginal Zone Lymphoma;; ND, Not Documented NR, Not Reached; pts, patients; R-CVEP, rituximab, cyclophosphamide, vorinostat, etoposide, and prednisone; R/R, Refractory/relapsed; y, years*.

The anti-PD-1 and anti-PD-L1 treatment approaches, coupled with other agents have produced somewhat disappointing results for recurrent DLBCL (74). Currently, inhibition of PD-1/PD-L1 is used in the clinical trial in combination CAR T cell therapy (NCT02926833 and NCT02706405) or recurrence after CAR-T cell therapy (NCT02650999).

CAR T therapies that target CD19 on B cell malignancies were effective against multiply relapsed DLBCL in initial trials and have confirmed their effectiveness at longer-term follow-up.

## Conclusion and Future Outlook

The development of drugs in lymphomas has undergone substantial changes in the last decade. An endeavor is ongoing to change conventional chemotherapy, with more targeted molecules directed against cell complexes and pathways that are explicitly related to lymphomagenesis. An overview of the ongoing trials is finally provided (Table 4). While mAbs have been the first trend of targeted therapies, there is now a new generation of biological agents, and more of them with an oral formulation, that takes full advantage of a superior understanding of lymphomagenesis. In addition, they have achieved outstanding results especially in subtypes with indolent behavior. Immune therapy with CIs and other models such as CAR-T cells and bispecific antibodies have shown promising results in mature B-cell lymphomas with aggressive behavior where other targeted agents have unfortunately demonstrated only modest improvements. Combined targeted therapy and chemotherapy will be a promising therapeutic strategy and is currently being exploited in ongoing trials (Tables 4, 5). However, early identification and appropriate management of toxicities should represent a significant issue since important adverse events have been reported, due to both on- and off-target effects, which have already been demonstrated to be unpredictable, leading to the early closure of some studies. Most notably, the occurrence of unforeseen immune events has highlighted the pitfalls of novel drugs emblematically, either as a single agent and/or in combination. Immune/inflammatory toxicities have been reported with checkpoint immunotherapy and combinations of PI3K/SYK inhibitors while hematologic toxicities are pronounced with the BCL-2 inhibitors and standard chemotherapy (245).

**Table 4:** Ongoing trials of immunotherapeutic agents in mature B cell neoplasms.

**Drug**	**Target**	**Histologic subtype**	**Study phase**	**Schedule**	**Setting (planned enrollment)**	**Current trials**
**ANTI-CD mAbs**						
Camidanlumab Tesirine	CD25	B-NHL	I	Single Agent, Adaptive Dose-Escalation Study	R/R (140 pts).	NCT02432235
Epratuzumab	CD22	HG-BCL	I/II	Randomized: 90Y-Epratuzumab wk 2 3 (days 8 & 15)	R/R (70 pts). Random Veltuzumab vs Epratuzumab	NCT01101581
Obinutuzumab	CD20	B-NHL	II	Randomized: Single Agent vs. O-ICE	R/R (25 pts)	NCT02393157
Ofatumumab	CD20	CLL/SLL	I	Dose finding	R/R (60 pts) In combination to rituximab. In addition to bendamustine	NCT02361346
Polatuzumab Vedotin	CD79b	DLBCL FL	Ib/II	Randomized: Pola+Rituximab vs. pola+Rituximab+Bendamustine	R/R. (314 pts) In combination to rituximab. In addition to bendamustine	NCT02257567
Ublituximab	CD20	B-NHL	I/II	450 mg followed by 600 mg, 900 or 1,200 mg in each cohort	R/R CD20 Directed Antibody Therapy	NCT01647971
**IMMUNE CHECKPOINT INHIBITORS**						
Atezolizumab	PD-L1	DLCBL	II	18 cycles followed by 12 mos of observation	R/R (114 pts) IPI-score ≥ 3 in patients to R/R R-CHOP	NCT03463057
Durvalumab	PD-L1	CLL/SLL	I/II	1,500 mg (IV) infusion on Day 1 of Cycles 1 through 13	R/R. (106 pts) In combination to Bendamustine, Lenalidomide and Rituximab in 4 arms	NCT02733042
Ipilimumab	CTLA-4	DLBCL	Ib/II	Ipilimumab mg/kg nivolumab 3 mg/kg	R/R (13 pts) whom are ineligible for ASCT.	NCT03305445
Nivolumab	PD-1	FL	I	240 mg IV q2-weekly for four cycle	Naive (39 pts).	NCT03245021
Pembrolizumab	PD-1	DLBCL	I/II	200 mg IV infusion (day 1), oral CXD101 20 mg twice daily.	R/R (45 pts). In combination to CXD101 HDAC inhibitor	NCT03873025
Pidilizumab	PD-1	iNHL	I/II	Dose safety	R/R (109 pts). In combination with ibrutinib. Three arms	NCT02401048
**(CAR**) **T-CELL THERAPY**						
lisocabtagene maraleucel	CAR-T	HG-BCL	II	Single dose intravenous	R/R (50 pts).	NCT03744676
Axicabtagene ciloleucel	CAR T-4-1BB	DLBCL	II	Single infusion of CAR-T post Fludarabina and cyclophosphamide.	High risk (40 pts).	NCT03761056
Axicabtagene ciloleucel	CAR T-4-1BB	DLBCL	II	single infusion of CAR-T	R/R (350 pts). Randomized vs. standard protocols (i.e., R-ICE)	NCT03391466
**BISPECIFIC ANTIBODIES**						
Blinatumomab	CD19/CD3	iNHL	II	Dose escalation 9–28 μg/day	R/R (28 pts). Single agent	NCT02811679
Mosunetuzumab (BTCT4465A)	CD20/CD3	iNHL and CLL/SLL	I/Ib	atezolizumab 1200 mg IV infusion in combination with Mosunetuzumab.	R/R (665 pts) in combination to Atezolumab	NCT02500407

*ASCT, Autologous Stem Cell Transplantation; B-NHL, B-NHL not otherwise specified; CLL/SLL, Chronic Lymphocytic Leukemia/Small Lymphocytic Leukemia; DLBCL, Diffuse Large B cell Lymphoma; FL, Follicular Lymphoma; HG-BCL, High Grade B-cell lymphoma; IGHV, Immunoglobulin G Heavy Variable chain; iNHL, indolent NHL; IV, intravenous; LBCL Large B-cell lymphoma; MCL, Mantle Cell Lymphoma; mo.s, months; MZL, Marginal Zone Lymphoma; ND, Not Documented NR, Not Reached; pts, patients; R- ICE, rituximab, cyclophosphamide, etoposide; R/R, Refractory/relapsed; y, years*.

**Table 5:** Ongoing trials of signal transduction pathway inhibitors in mature B cell neoplasms.

**Drug**	**Target**	**Histologic subtype**	**Study phase**	**Schedule**	**Setting (planned enrollment)**	**Current trials**
**BCR INHIBITORS**						
Acalabrutinib	BTK	HG-BCL	I	IV infusion days 1, 3, 5	R/R (42 pts)	NCT03527147
Ibrutinib	BTK	MCL	III	1 tablet/day	Randomized in combination to Venetoclax (287 pts)	NCT03112174
Ibrutinib	BTK	DLBCL	Ib Dose Finding	1 tablet/day until disease progression	R/R (30 pts) in combination to Rituximab and Venetoclax	NCT03136497
LOXO-305	BTK C481 mutation	CLL/SLL	I/II	25 mg/day	R/R with C481 mutation in *BTK* gene.	NCT03740529
Copanlisib	PI3K	B-NHLnos	Ib/II	Days 1, 8, and 15 of a 28-day cycle	R/R (25 pts)	NCT02342665
Duvelisib	PI3Kδ+γ	CLL/SLL	I/II	Orally twice daily	R/R (47 pts) in combination with Venetoclax	NCT03534323
Idelalisib	PI3K δ	CLL/SLL iNHL	II	1 tablet/day (cycle 21 day)	R/R (68 pts). Combination to Pembrolizumab	NCT02332980
Cerdulatinib (PRT062070)	SYKJAK 1-2	FL, DLBCL	I/IIa	Dose finding	R/R (283 pts)	NCT01994382
TAK659	SYK/FLT3	FL, MZL	I	60–80 mg/day	R/R (47 pts). Single agent	NCT03238651
**PROTEASOME INHIBITORS**						
Bortezomib	PIs	B-NHLnos	I/II	MTD	R/R (56 pts). Combination to Gemcitabime and Rituximab	NCT00863369
Ixazomib	20S subunit	iNHL	II	once weekly × 4 wk	naïve iNHL (36 pts). In addition to Rituximab sd	NCT02339922
**mTor- INHIBITORS**						
Temsirolimus	mTor	Lymphoblastic Lymphoma	I	Day 1-8 IV	R/R (30 pts). in combination to Etoposide and Cyclophosphamide	NCT01614197
**BCL2 INHIBITOR**						
Venetoclax	BH3 domain	DLBCL	Ib	1 tablet/day (cycle 28 day)	R/R (30 pts). In combination with Rituximab with 17p deletion	NCT03136497
**HDAC INHIBITORS**						
CUDC-907	Class I and II+ PI3K	DLBCL	II	ND	R/R (200 pts) with Myc alteration	NCT02674750
Mocetinostat (MGCD0103)	Class I and IV	DLBCL	II	70 mg/3 times per week on a 28 day	R/R (7 pts) with mutations of Acetyltransferase Genes	NCT02282358
Panobinostat	Class I, II and IV	DLBCL	II	30 mg/day	R/R (42 pts). Randomized with or without Rituximab	NCT01238692
Tazemetostat	EZH2	DLBCL FL	I/II	Dose escalation	Single agent (420 pts)	NCT01897571
Vorinostat	Class I and II	DLBCL, FL	I	Days 1–5 and 8–12. Cycle 21 days	R/R (60 pts). In combination to Pembrolizumab	NCT03150329

*ASCT, Autologous Stem Cell Transplantation; B-NHL, B-NHL not otherwise specified; CLL/SLL, Chronic Lymphocytic Leukemia/Small Lymphocytic Leukemia; DLBCL, Diffuse Large B cell Lymphoma; FL, Follicular Lymphoma; HG-BCL, High Grade B-cell lymphoma; IGHV, Immunoglobulin G Heavy Variable chain; iNHL, indolent NHL; IV, intravenous; LBCL Large B-cell lymphoma; MCL, Mantle Cell Lymphoma; mo.s, months; MZL, Marginal Zone Lymphoma; ND, Not Documented NR, Not Reached; pts, patients; R-CVEP, rituximab, cyclophosphamide, vorinostat, etoposide, and prednisone; R/R, Refractory/relapsed; y, years; MTD, maximum tolerated dose; wk, week; sd, standard dosage*.

With the current knowledge of target therapies, each patient's cancer biology may be driven to the best cancer treatment.

## Author Contributions

SC and RaD wrote the manuscript. SC, MB, and PR selected bibliography. SC and GR prepared Figure 1. PV and SM prepared the Table 1. RoD and AP over-reviewed the manuscript.

### Conflict of Interest Statement

The authors declare that the research was conducted in the absence of any commercial or financial relationships that could be construed as a potential conflict of interest.

## References

[1] Swerdlow SH Campo E Pileri SA Harris NL Stein H Siebert R. The 2016 revision of the World Health Organization classification of lymphoid neoplasms. Blood. (2016) 127:2375–90. 10.1182/blood-2016-01-643569 26980727 PMC4874220

[2] Rizvi MA Evens AM Tallman MS Nelson BP Rosen ST. T-cell non-Hodgkin lymphoma. Blood. (2006) 107:1255–64. 10.1182/blood-2005-03-1306 16210342

[3] Jiang M Bennani NN Feldman AL. Lymphoma classification update: B-cell non-Hodgkin lymphomas. Expert Rev Hematol. (2017) 10:405–15. 10.1080/17474086.2017.1318053 28395545

[4] Gisselbrecht C Van Den Neste E. How I manage patients with relapsed/refractory diffuse large B cell lymphoma. Br J Haematol. (2018) 182:633–43. 10.1111/bjh.15412 29808921 PMC6175435

[5] El-Mallawany NK Cairo MS. Advances in the diagnosis and treatment of childhood and adolescent B-cell non-Hodgkin lymphoma. Clin Adv Hematol Oncol. (2015) 13:113–23. 25774481

[6] Mei M Chen R. How to approach a Hodgkin lymphoma patient with relapse after autologous SCT: allogeneic SCT. Clin Lymphoma Myeloma Leuk. (2018) 18:26–33. 10.1016/j.clml.2017.11.003 29233742

[7] Shanbhag S Ambinder RF. Hodgkin lymphoma: a review and update on recent progress. CA Cancer J Clin. (2018) 68:116–32. 10.3322/caac.21438 29194581 PMC5842098

[8] Biccler JL El-Galaly TC Bogsted M Jorgensen J de Nully Brown P Poulsen CB. Clinical prognostic scores are poor predictors of overall survival in various types of malignant lymphomas. Leuk Lymphoma. (2018). 10.1080/10428194.2018.1540044. [Epub ahead of print]. 30424711

[9] Barth MJ Minard-Colin V. Novel targeted therapeutic agents for the treatment of childhood, adolescent and young adult non-Hodgkin lymphoma. Br J Haematol. (2019). 10.1111/bjh.15783. [Epub ahead of print]. 30701541

[10] Al Juhaishi T Yazbeck V. Choosing the right pharmacotherapy for non-Hodgkin's lymphoma: does one size fit all? Expert Opin Pharmacother. (2019) 20:773–5. 10.1080/14656566.2019.1582643 30793985

[11] Gerecitano J. The future of small molecule inhibitors in lymphoma. Curr Oncol Rep. (2009) 11:378–85. 10.1007/s11912-009-0051-1 19679013

[12] Ma H Sawas A. Combining biology and chemistry for a new take on chemotherapy: antibody-drug conjugates in hematologic malignancies. Curr Hematol Malig Rep. (2018) 13:555–69. 10.1007/s11899-018-0485-3 30362019

[13] Ladetto M Buske C Hutchings M Dreyling M Gaidano G Le Gouill S. ESMO consensus conference on malignant lymphoma: general perspectives and recommendations for prognostic tools in mature B-cell lymphomas and chronic lymphocytic leukaemia. Ann Oncol. (2018) 29:525. 10.1093/annonc/mdx061 28368466

[14] Lange J Lenz G Burkhardt B. Mature aggressive B-cell lymphoma across age groups - molecular advances and therapeutic implications. Expert Rev Hematol. (2017) 10:123–35. 10.1080/17474086.2017.1271318 27936978

[15] Wang TP Scott JH Barta SK. The evolving role of targeted biological agents in the management of indolent B-cell lymphomas. Ther Adv Hematol. (2017) 8:329–44. 10.1177/2040620717738740 29204260 PMC5703116

[16] Merryman RW Armand P Wright KT Rodig SJ. Checkpoint blockade in Hodgkin and non-Hodgkin lymphoma. Blood Adv. (2017) 1:2643–54. 10.21037/aol.2017.08.03 29296917 PMC5728646

[17] Horn H Staiger AM Ott G. New targeted therapies for malignant lymphoma based on molecular heterogeneity. Expert Rev Hematol. (2017) 10:39–51. 10.1080/17474086.2017.1268046 27918211

[18] Rosenthal A. Small molecule inhibitors in chronic lymphocytic lymphoma and B cell non-Hodgkin lymphoma. Curr Hematol Malig Rep. (2017) 12:207–16. 10.1007/s11899-017-0383-0 28439761

[19] von Keudell G Younes A. Novel therapeutic agents for relapsed classical Hodgkin lymphoma. Br J Haematol. (2019) 184:105–12. 10.1111/bjh.15695 30536386

[20] Marron TU Kalac M Brody J. An update on the use of immunotherapy in the treatment of lymphoma. Curr Hematol Malig Rep. (2017) 12:282–9. 10.1007/s11899-017-0396-8 28735365

[21] Wang M Liu Y Cheng Y Wei Y Wei X. Immune checkpoint blockade and its combination therapy with small-molecule inhibitors for cancer treatment. Biochim Biophys Acta Rev Cancer. (2018) 1871:199–224. 10.1016/j.bbcan.2018.12.002 30605718

[22] Rossig C. CAR T cell immunotherapy in hematology and beyond. Clin Immunol. (2018) 186:54–8. 10.1016/j.clim.2017.09.016 28923441

[23] Copelan EA. CAR T-cell therapy in non-Hodgkin lymphoma patients. Oncology. (2019) 33:73–4. 30784033

[24] Nair R Neelapu SS. The promise of CAR T-cell therapy in aggressive B-cell lymphoma. Best Pract Res Clin Haematol. (2018) 31:293–8. 10.1016/j.beha.2018.07.011 30213399 PMC6594830

[25] Pishko A Nasta SD. The role of novel immunotherapies in non-Hodgkin lymphoma. Transl Cancer Res. (2017) 6:93–103. 10.21037/tcr.2017.01.08 28955654 PMC5612371

[26] Marrocco I Romaniello D Yarden Y. Cancer immunotherapy: the dawn of antibody cocktails. Methods Mol Biol. (2019) 1904:11–51. 10.1007/978-1-4939-8958-4_2 30539465

[27] Samarasinghe SA Shao Y Huang PJ Pishko M Chu KH Kameoka J. Fabrication of bacteria environment cubes with dry lift-off fabrication process for enhanced nitrification. PLoS ONE. (2016) 11:e0165839. 10.1371/journal.pone.0165839 27812154 PMC5094588

[28] Sawas A Farber CM Schreeder MT Khalil MY Mahadevan D Deng CC. A Phase 1/2 trial of ublituximab, a novel anti-CD20 monoclonal antibody, in patients with B-cell non-Hodgkin lymphoma or chronic lymphocytic leukaemia previously exposed to rituximab. Br J Haematol. (2017) 177:243–53. 10.1111/bjh.14534 28220479 PMC5412890

[29] CLL: Alemtuzumab increases progression-free survival Onkologie. (2007) 30:217.

[30] Alinari L Lapalombella R Andritsos L Baiocchi RA Lin TS Byrd JC. Alemtuzumab (Campath-1H) in the treatment of chronic lymphocytic leukemia. Oncogene. (2007) 26:3644–53. 10.1038/sj.onc.1210380 17530018

[31] Al-Sawaf O Fischer K Herling CD Ritgen M Bottcher S Bahlo J. Alemtuzumab consolidation in chronic lymphocytic leukaemia: a Phase I/II multicentre trial. Eur J Haematol. (2017) 98:254–62. 10.1111/ejh.12825 27862308

[32] Vaklavas C Forero-Torres A Safety and efficacy of brentuximab vedotin in patients with Hodgkin lymphoma or systemic anaplastic large cell lymphoma. Ther Adv Hematol. (2012) 3:209–25. 10.1177/2040620712443076 23606932 PMC3627331

[33] Tomassetti S Herrera AF. Update on the role of brentuximab vedotin in classical Hodgkin lymphoma. Ther Adv Hematol. (2018) 9:261–72. 10.1177/2040620718786833 30210755 PMC6130098

[34] Donato EM Fernandez-Zarzoso M Hueso JA de la Rubia J. Brentuximab vedotin in Hodgkin lymphoma and anaplastic large-cell lymphoma: an evidence-based review. Onco Targets Ther. (2018) 11:4583–90. 10.2147/OTT.S141053 30122950 PMC6084082

[35] Berger GK McBride A Lawson S Royball K Yun S Gee K. Brentuximab vedotin for treatment of non-Hodgkin lymphomas: a systematic review. Crit Rev Oncol Hematol. (2017) 109:42–50. 10.1016/j.critrevonc.2016.11.009 28010897 PMC5218629

[36] Bhatt G Maddocks K Christian B. CD30 and CD30-targeted therapies in Hodgkin lymphoma and other B cell lymphomas. Curr Hematol Malig Rep. (2016) 11:480–91. 10.1007/s11899-016-0345-y 27613003

[37] Bhatt S Ashlock BM Natkunam Y Sujoy V Chapman JR Ramos JC. CD30 targeting with brentuximab vedotin: a novel therapeutic approach to primary effusion lymphoma. Blood. (2013) 122:1233–42. 10.1182/blood-2013-01-481713 23838350 PMC3744990

[38] Flynn MJ Zammarchi F Tyrer PC Akarca AU Janghra N Britten CE. ADCT-301, a Pyrrolobenzodiazepine (PBD) dimer-containing Antibody-Drug Conjugate (ADC) targeting CD25-expressing hematological malignancies. Mol Cancer Ther. (2016) 15:2709–21. 10.1158/1535-7163.MCT-16-0233 27535974

[39] Flynn MJ van Berkel P Zammarchi F Levy JN Tiberghien A Masterson LA Pre-clinical activity of Adct-301, a novel Pyrrolobenzodiazepine (PBD) dimer-containing Antibody Drug Conjugate (ADC) targeting CD25-expressing hematological malignancies. Blood. (2014) 124:4491. 10.1158/1535-7163.MCT-16-0233 27535974

[40] Satwani P Perkins S Kinney M Davenport V Sposto R Abromowitch M CD52 and CD25 are highly expressed in childhood non-Hodgkin's lymphoma and may be excellent targets for immunotherapy with alemtuzumab and/or denileukin diftitox, respectively. Ann Oncol. (2005) 16:133.

[41] Advani R Forero-Torres A Furman RR Rosenblatt JD Younes A Ren H Phase I study of the humanized anti-CD40 monoclonal antibody dacetuzumab in refractory or recurrent non-Hodgkin's lymphoma. J Clin Oncol. (2009) 27:4371–7. 10.1200/JCO.2008.21.3017 19636010

[42] Burington B Advani R Shi XY Yue P Lau J Yu SF A gene signature predicts sensitivity to the partial CD40 agonist, dacetuzumab (SGN-40), in patients with diffuse large B-cell lymphoma. Cancer Res. (2009) 69.

[43] Gowda AC Zhao XB Cheney C Mehter N Lozanski G Lin TS Humanized anti CD-40 antibody SGN-40 effectively induces cytotoxicity against chronic lymphocytic leukemia (CLL) cells through antibody mediated cytotoxicity and demonstrates modest biologic evidence of CD40 activation. Blood. (2005) 106:832a-a.

[44] Byrd JC Kipps TJ Flinn IW Cooper M Odenike O Bendiske J. Phase I study of the anti-CD40 humanized monoclonal antibody lucatumumab (HCD122) in relapsed chronic lymphocytic leukemia. Leuk Lymphoma. (2012) 53:2136–42. 10.3109/10428194.2012.681655 22475052 PMC3808981

[45] Cheson BD Trneny M Bouabdallah K Dueck G Gribben J Lugtenburg PJ Obinutuzumab plus bendamustine followed by obinutuzumab maintenance prolongs overall survival compared with bendamustine alone in patients with rituximab-refractory indolent non-Hodgkin lymphoma: updated results of the GADOLIN Study. Blood. (2016) 128:615.27288518

[46] Teeling JL Mackus WJM Wiegman LJJM Van den Brakel JHN Beers SA French RR. The biological activity of human CD20 monoclonal antibodies is linked to unique epitopes on CD20. J Immunol. (2006) 177:362–71. 10.4049/jimmunol.177.1.362 16785532

[47] Teeling JL French RR Cragg MS van den Brakel J Pluyter M Huang H. Characterization of new human CD20 monoclonal antibodies with potent cytolytic activity against non-Hodgkin lymphomas. Blood. (2004) 104:1793–800. 10.1182/blood-2004-01-0039 15172969

[48] Maloney DG Fukuhara N Ogura M Larouche JF Tournilhac O Coleman M A Phase III study of ofatumumab vs rituximab in indolent B-cell non-Hodgkin lymphoma relapsed after rituximab containing therapy (homer): results of the interim analysis. Haematologica. (2016) 101:102.

[49] Palanca-Wessels MC Flinn IW Sehn LH Patel M Sangha R Czuczman MS. A Phase I study of the anti-CD79b Antibody-Drug Conjugate (ADC) DCDS4501A targeting CD79b in relapsed or refractory B-cell non-Hodgkin's lymphoma (NHL). Blood. (2012) 120:56. 22611150

[50] Sehn LH Herrera AF Matasar MJ Kamdar M Assouline S Hertzberg M Polatuzumab vedotin (pola) plus Bendamustine (B) with Rituximab (R) or Obinutuzumab (G) in Relapsed/Refractory (R/R) Diffuse Large B-Cell Lymphoma (DLBCL): updated results of a Phase (Ph) Ib/II study. Blood. (2018) 132:1683 10.1182/blood-2018-99-118551

[51] Gajdosik Z Polatuzumab vedotin anti-CD79b antibody-drug conjugate treatment of hematologic malignancies. Drugs Future. (2016) 41:411–6. 10.1358/dof.2016.041.07.2505619

[52] Herrera AF Matasar MJ Assouline S Kamdar M Mehta A Fleury I Polatuzumab vedotin combined with Bendamustine (B) and Rituximab (R) or Obinutuzumab (G) in patients with Relapsed or Refractory (R/R) Follicular Lymphoma (FL) or Diffuse Large B-Cell Lymphoma (DLBCL): preliminary results of a Phase Ib/II dose-escalation study. Blood. (2016) 128.27827828

[53] Forero-Torres A Kolibaba KS Lamy T Jones S Lee C Sharman J Polatuzumab vedotin combined with Obinutuzumab, Cyclophosphamide, Doxorubicin, and Prednisone (G-CHP) for patients with previously untreated Diffuse Large B-Cell Lymphoma (DLBCL): preliminary results of a Phase Ib/II dose-escalation study. Blood. (2016) 128:1856.

[54] Bartlett NL Chen AI Kolibaba KS Lamy T Jones S Hirata J Polatuzumab vedotin combined with Rituximab, Cyclophosphamide, Doxorubicin, and Prednisone (R-CHP) for patients with previously untreated Diffuse Large B-Cell Lymphoma (DLBCL): preliminary results of a Phase Ib dose-escalation. Blood. (2015) 126:2726.

[55] Marshall MJE Stopforth RJ Cragg MS. Therapeutic antibodies: what have we learnt from targeting CD20 and where are we going? Front Immunol. (2017) 8:1245. 10.3389/fimmu.2017.01245 29046676 PMC5632755

[56] Salles G Barrett M Foa R Maurer J O'Brien S Valente N. Rituximab in B-cell hematologic malignancies: a review of 20 years of clinical experience. Adv Ther. (2017) 34:2232–73. 10.1007/s12325-017-0612-x 28983798 PMC5656728

[57] Kolibaba K Burke JM Brooks HD Mahadevan D Melear J Farber CM Ublituximab (TG-1101), a novel glycoengineered anti-CD20 monoclonal antibody, in combination with ibrutinib is highly active in patients with relapsed and/or refractory mantle cell lymphoma; results of a Phase II trial. Blood. (2015) 126:3980.

[58] Carter P. Improving the efficacy of antibody-based cancer therapies. Nat Rev Cancer. (2001) 1:118–29. 10.1038/35101072 11905803

[59] Hu B Jacobs R Ghosh N. Checkpoint inhibitors Hodgkin lymphoma and non-Hodgkin lymphoma. Curr Hematol Malig Rep. (2018) 13:543–54. 10.1007/s11899-018-0484-4 30338457

[60] Ansell SM. Harnessing the power of the immune system in non-Hodgkin lymphoma: immunomodulators, checkpoint inhibitors, and beyond. Hematol Am Soc Hematol Educ Program. 2017:618–21. 10.1182/asheducation-2017.1.618 29222312 PMC6142557

[61] Savage KJ Steidl C. Immune checkpoint inhibitors in Hodgkin and non-Hodgkin lymphoma: how they work and when to use them. Expert Rev Hematol. (2016) 9:1007–9. 10.1080/17474086.2016.1242404 27677541

[62] Sun L Chen LX Li H. Checkpoint-modulating immunotherapies in tumor treatment: targets, drugs, and mechanisms. Int Immunopharmacol. (2019) 67:160–75. 10.1016/j.intimp.2018.12.006 30553199

[63] Ansell SM PD-1 targeted therapy in lymphomas. Br J Haematol. (2018) 182:13.

[64] Ansell SM Lesokhin AM Borrello I Halwani A Scott EC Gutierrez M PD-1 blockade with nivolumab in relapsed or refractory Hodgkin's lymphoma. N Engl J Med. (2015) 372:311–9. 10.1056/NEJMoa1411087 25482239 PMC4348009

[65] Ding W Laplant B Witzig TE Johnston PB Colgan JP Rech KL PD-1 blockade with pembrolizumab in relapsed low grade non-Hodgkin lymphoma. Blood. (2017) 130:4055.

[66] Villasboas JC Ansell SM Witzig TE. Targeting the PD-1 pathway in patients with relapsed classic Hodgkin lymphoma following allogeneic stem cell transplant is safe and effective. Oncotarget. (2016) 7:13260–4. 10.18632/oncotarget.7177 26848626 PMC4914357

[67] Garon EB Rizvi NA Hui RN Leighl N Balmanoukian AS Eder JP. Pembrolizumab for the treatment of non-small-cell lung cancer. N Engl J Med. (2015) 372:2018–28. 10.1056/NEJMoa1501824 25891174

[68] Ninomiya K Hotta K. Pembrolizumab for the first-line treatment of non-small cell lung cancer. Expert Opin Biol Ther. (2018) 18:1015–21. 10.1080/14712598.2018.1522300 30207786

[69] Motzer RJ Rini BI McDermott DF Redman BG Kuzel TM Harrison MR. Nivolumab for metastatic renal cell carcinoma: results of a randomized Phase II trial. J Clin Oncol. (2015) 33:1430–7. 10.1200/JCO.2014.59.0703 25452452 PMC4806782

[70] Ishida Y Agata Y Shibahara K Honjo T. Induced expression of PD-1, a novel member of the immunoglobulin gene superfamily, upon programmed cell death. EMBO J. (1992) 11:3887–95. 10.1002/j.1460-2075.1992.tb05481.x 1396582 PMC556898

[71] Matsuki E Younes A. Checkpoint inhibitors and other immune therapies for Hodgkin and non-Hodgkin lymphoma. Curr Treat Options Oncol. (2016) 17:31. 10.1007/s11864-016-0401-9 27193488 PMC5578701

[72] Linsley PS Golstein P. Lymphocyte activation: T-cell regulation by CTLA-4. Curr Biol. (1996) 6:398–400. 10.1016/S0960-9822(02)00506-7 8723343

[73] Linsley PS Bradshaw J Greene J Peach R Bennett KL Mittler RS. Intracellular trafficking of CTLA-4 and focal localization towards sites of TCR engagement. Immunity. (1996) 4:535–43. 10.1016/S1074-7613(00)80480-X 8673700

[74] Linsley PS Greene JL Brady W Bajorath J Ledbetter JA Peach R. Human B7-1 (CD80) and B7-2 (CD86) bind with similar avidities but distinct kinetics to CD28 and CTLA-4 receptors. Immunity. (1994) 1:793–801. 10.1016/S1074-7613(94)80021-9 7534620

[75] Xu-Monette ZY Zhou J Young KH. PD-1 expression and clinical PD-1 blockade in B-cell lymphomas. Blood. (2018) 131:68–83. 10.1182/blood-2017-07-740993 29118007 PMC5755041

[76] Alsaab HO Sau S Alzhrani R Tatiparti K Bhise K Kashaw SK. PD-1 and PD-L1 checkpoint signaling inhibition for cancer immunotherapy: mechanism, combinations, and clinical outcome. Front Pharmacol. (2017) 8:561. 10.3389/fphar.2017.00561 28878676 PMC5572324

[77] Witkowska M Smolewski P. Immune checkpoint inhibitors to treat malignant lymphomas. J Immunol Res. (2018) 2018:1982423. 10.1155/2018/1982423 29850620 PMC5925139

[78] Lee HT Lee JY Lim H Lee SH Moon YJ Pyo HJ. Molecular mechanism of PD-1/PD-L1 blockade via anti-PD-L1 antibodies atezolizumab and durvalumab. Sci Rep. (2017) 7:5532. 10.1038/s41598-017-06002-8 28717238 PMC5514103

[79] Khouri IF Fernandez Curbelo I Turturro F Jabbour EJ Milton DR Bassett RL Jr. Ipilimumab plus lenalidomide after allogeneic and autologous stem cell transplantation for patients with lymphoid malignancies. Clin Cancer Res. (2018) 24:1011–8. 10.1158/1078-0432.CCR-17-2777 29246938 PMC5844825

[80] Younes A Santoro A Shipp M Zinzani PL Timmerman JM Ansell S. Nivolumab for classical Hodgkin's lymphoma after failure of both autologous stem-cell transplantation and brentuximab vedotin: a multicentre, multicohort, single-arm Phase 2 trial. Lancet Oncol. (2016) 17:1283–94. 10.1016/S1470-2045(16)30167-X 27451390 PMC5541855

[81] Lesokhin AM Ansell SM Armand P Scott EC Halwani A Gutierrez M. Nivolumab in patients with relapsed or refractory hematologic malignancy: preliminary results of a Phase Ib study. J Clin Oncol. (2016) 34:2698–704. 10.1200/JCO.2015.65.9789 27269947 PMC5019749

[82] Moskowitz CH Ribrag V Michot JM Martinelli G Zinzani PL Gutierrez M PD-1 blockade with the monoclonal antibody pembrolizumab (MK-3475) in patients with classical Hodgkin lymphoma after brentuximab vedotin failure: preliminary results from a Phase 1b study (KEYNOTE-013). Blood. (2014) 124:290.

[83] Ding W LaPlant BR Call TG Parikh SA Leis JF He R Pembrolizumab in patients with CLL and Richter transformation or with relapsed CLL. Blood. (2017) 129:3419–27. 10.1182/blood-2017-02-765685 28424162 PMC5492091

[84] Menter T Bodmer-Haecki A Dirnhofer S Tzankov A. Evaluation of the diagnostic and prognostic value of PDL1 expression in Hodgkin and B-cell lymphomas. Hum Pathol. (2016) 54:17–24. 10.1016/j.humpath.2016.03.005 27045512

[85] Silverman E. Kymriah: A sign of more difficult decisions to come. Manag Care. (2018) 27:17. 29763402

[86] Morrow T. Novartis's Kymriah: harnessing immune system comes with worry about reining in costs. Manag Care. (2017) 26:28–30. 29068298

[87] Tisagenlecleucel (Kymriah) for ALL Med Lett Drugs Ther. (2017) 59:177–8. 29039821

[88] Horton HM Bernett MJ Pong E Peipp M Karki S Chu SY. Potent *in vitro* and *in vivo* activity of an Fc-engineered anti-CD19 monoclonal antibody against lymphoma and leukemia. Cancer Res. (2008) 68:8049–57. 10.1158/0008-5472.CAN-08-2268 18829563

[89] Boyiadzis MM Dhodapkar MV Brentjens RJ Kochenderfer JN Neelapu SS Maus MV. Chimeric antigen receptor (CAR) T therapies for the treatment of hematologic malignancies: clinical perspective and significance. J Immunother Cancer. (2018) 6:137. 10.1186/s40425-018-0460-5 30514386 PMC6278156

[90] Axicabtagene ciloleucel (Yescarta) for B-cell lymphoma Med Lett Drugs Ther. (2018) 60:e122–3. 30036350

[91] Brudno JN Kochenderfer JN. Recent advances in CAR T-cell toxicity: mechanisms, manifestations and management. Blood Rev. (2019) 34:45–55. 10.1016/j.blre.2018.11.002 30528964 PMC6628697

[92] Brudno JN Kochenderfer JN. Toxicities of chimeric antigen receptor T cells: recognition and management. Blood. (2016) 127:3321–30. 10.1182/blood-2016-04-703751 27207799 PMC4929924

[93] Zhang LN Song Y Liu D. CD19 CAR-T cell therapy for relapsed/refractory acute lymphoblastic leukemia: factors affecting toxicities and long-term efficacies. J Hematol Oncol. (2018) 11:41. 10.1186/s13045-018-0593-5 29544528 PMC5855988

[94] Gross G Eshhar Z. Therapeutic potential of T cell Chimeric Antigen Receptors (CARs) in cancer treatment: counteracting off-tumor toxicities for safe CAR T cell therapy. Annu Rev Pharmacol Toxicol. (2016) 56:59–83. 10.1146/annurev-pharmtox-010814-124844 26738472

[95] Hirayama AV Turtle CJ. Toxicities of CD19 CAR-T cell immunotherapy. Am J Hematol. (2019) 94:S42–9. 10.1002/ajh.25445 30784102

[96] Lameris R de Bruin RC Schneiders FL van Bergen en Henegouwen PM Verheul HM de Gruijl TD. Bispecific antibody platforms for cancer immunotherapy. Crit Rev Oncol Hematol. (2014) 92:153–65. 10.1016/j.critrevonc.2014.08.003 25195094

[97] Kontermann RE Brinkmann U. Bispecific antibodies. Drug Discov Today. (2015) 20:838–47. 10.1016/j.drudis.2015.02.008 25728220

[98] Fan G Wang Z Hao M Li J. Bispecific antibodies and their applications. J Hematol Oncol. (2015) 8:130. 10.1186/s13045-015-0227-0 26692321 PMC4687327

[99] Viardot A Bargou R. Bispecific antibodies in haematological malignancies. Cancer Treat Rev. (2018) 65:87–95. 10.1016/j.ctrv.2018.04.002 29635163

[100] Nazarian AA Archibeque IL Nguyen YH Wang P Sinclair AM Powers DA. Characterization of bispecific T-cell Engager (BiTE) antibodies with a high-capacity T-cell dependent cellular cytotoxicity (TDCC) assay. J Biomol Screen. (2015) 20:519–27. 10.1177/1087057114561405 25477202

[101] Yuraszeck T Kasichayanula S Benjamin JE. Translation and clinical development of bispecific T-cell engaging antibodies for cancer treatment. Clin Pharmacol Ther. (2017) 101:634–45. 10.1002/cpt.651 28182247 PMC5763312

[102] Velasquez MP Bonifant CL Gottschalk S. Redirecting T cells to hematological malignancies with bispecific antibodies. Blood. (2018) 131:30–8. 10.1182/blood-2017-06-741058 29118005 PMC5755042

[103] Ansell SM Chen RW Forero-Torres A Armand P Lossos IS Reeder CB A Phase 1 study investigating the combination of AFM13 and the monoclonal anti-PD-1 antibody pembrolizumab in patients with relapsed/refractory Hodgkin lymphoma after brentuximab vedotin failure: data from the dose escalation part of the study. Blood. (2017) 130:1522.

[104] Xu L Wang S Li J Li B. CD47/SIRPalpha blocking enhances CD19/CD3-bispecific T cell engager antibody-mediated lysis of B cell malignancies. Biochem Biophys Res Commun. (2019) 509:739–45. 10.1016/j.bbrc.2018.12.175 30611570

[105] Kaplan JB Grischenko M Giles FJ. Blinatumomab for the treatment of acute lymphoblastic leukemia. Invest New Drugs. (2015) 33:1271–9. 10.1007/s10637-015-0289-4 26383529

[106] Johnson S Burke S Huang L Gorlatov S Li H Wang W. Effector cell recruitment with novel Fv-based dual-affinity re-targeting protein leads to potent tumor cytolysis and *in vivo* B-cell depletion. J Mol Biol. (2010) 399:436–49. 10.1016/j.jmb.2010.04.001 20382161

[107] Moore PA Zhang W Rainey GJ Burke S Li H Huang L. Application of dual affinity retargeting molecules to achieve optimal redirected T-cell killing of B-cell lymphoma. Blood. (2011) 117:4542–51. 10.1182/blood-2010-09-306449 21300981

[108] Rader C. DARTs take aim at BiTEs. Blood. (2011) 117:4403–4. 10.1182/blood-2011-02-337691 21527536

[109] Goebeler ME Knop S Viardot A Kufer P Topp MS Einsele H. Bispecific T-Cell Engager (BiTE) antibody construct blinatumomab for the treatment of patients with relapsed/refractory non-Hodgkin lymphoma: final results from a Phase I study. J Clin Oncol. (2016) 34:1104–11. 10.1200/JCO.2014.59.1586 26884582

[110] Viardot A Goebeler ME Hess G Neumann S Pfreundschuh M Adrian N. Phase 2 study of the bispecific T-cell engager (BiTE) antibody blinatumomab in relapsed/refractory diffuse large B-cell lymphoma. Blood. (2016) 127:1410–6. 10.1182/blood-2015-06-651380 26755709 PMC4797019

[111] Siddiqi T Rosen ST. Novel biologic agents for non-Hodgkin lymphoma and chronic lymphocytic leukemia-part 2: adoptive cellular immunotherapy, small-molecule inhibitors, and immunomodulation. Oncology. (2015) 29:299–308. 25920929

[112] Rhodes J Landsburg DJ. Small-molecule inhibitors for the treatment of diffuse large B cell lymphoma. Curr Hematol Malig Rep. (2018) 13:356–68. 10.1007/s11899-018-0467-5 30112707

[113] Monroe JG. ITAM-mediated tonic signalling through pre-BCR and BCR complexes. Nat Rev Immunol. (2006) 6:283–94. 10.1038/nri1808 16557260

[114] Vanhaesebroeck B Leevers SJ Panayotou G Waterfield MD. Phosphoinositide 3-kinases: a conserved family of signal transducers. Trends Biochem Sci. (1997) 22:267–72. 10.1016/S0968-0004(97)01061-X 9255069

[115] Johnson SA Pleiman CM Pao L Schneringer J Hippen K Cambier JC. Phosphorylated immunoreceptor signaling motifs (ITAMs) exhibit unique abilities to bind and activate Lyn and Syk tyrosine kinases. J Immunol. (1995) 155:4596–603. 7594458

[116] Takata M Sabe H Hata A Inazu T Homma Y Nukada T. Tyrosine kinases Lyn and Syk regulate B cell receptor-coupled Ca2+ mobilization through distinct pathways. EMBO J. (1994) 13:1341–9. 10.1002/j.1460-2075.1994.tb06387.x 8137818 PMC394950

[117] Hallek M Fischer K Fingerle-Rowson G Fink AM Busch R Mayer J. Addition of rituximab to fludarabine and cyclophosphamide in patients with chronic lymphocytic leukaemia: a randomised, open-label, Phase 3 trial. Lancet. (2010) 376:1164–74. 10.1016/S0140-6736(10)61381-5 20888994

[118] Pettitt AR Jackson R Carruthers S Dodd J Dodd S Oates M. Alemtuzumab in combination with methylprednisolone is a highly effective induction regimen for patients with chronic lymphocytic leukemia and deletion of TP53: final results of the national cancer research institute CLL206 trial. J Clin Oncol. (2012) 30:1647–55. 10.1200/JCO.2011.35.9695 22493413

[119] Jaglowski SM Jones JA Nagar V Flynn JM Andritsos LA Maddocks KJ. Safety and activity of BTK inhibitor ibrutinib combined with ofatumumab in chronic lymphocytic leukemia: a Phase 1b/2 study. Blood. (2015) 126:842–50. 10.1182/blood-2014-12-617522 26116658 PMC4536539

[120] Burger JA Keating MJ Wierda WG Hartmann E Hoellenriegel J Rosin NY. Safety and activity of ibrutinib plus rituximab for patients with high-risk chronic lymphocytic leukaemia: a single-arm, Phase 2 study. Lancet Oncol. (2014) 15:1090–9. 10.1016/S1470-2045(14)70335-3 25150798 PMC4174348

[121] Byrd JC Harrington B O'Brien S Jones JA Schuh A Devereux S. Acalabrutinib (ACP-196) in relapsed chronic lymphocytic leukemia. N Engl J Med. (2016) 374:323–32. 10.1056/NEJMoa1509981 26641137 PMC4862586

[122] Tam C Grigg AP Opat S Ku M Gilbertson M Anderson MA The BTK inhibitor, Bgb-3111, is safe, tolerable, and highly active in patients with relapsed/ refractory B-cell malignancies: initial report of a Phase 1 first-in-human trial. Blood. (2015) 126:832.

[123] Walter HS Rule SA Dyer MJ Karlin L Jones C Cazin B. A Phase 1 clinical trial of the selective BTK inhibitor ONO/GS-4059 in relapsed and refractory mature B-cell malignancies. Blood. (2016) 127:411–9. 10.1182/blood-2015-08-664086 26542378 PMC4731845

[124] Brown JR Harb WA Hill BT Gabrilove J Sharman JP Schreeder MT. Phase I study of single-agent CC-292, a highly selective Bruton's tyrosine kinase inhibitor, in relapsed/refractory chronic lymphocytic leukemia. Haematologica. (2016) 101:e295–8. 10.3324/haematol.2015.140806 27151992 PMC5004476

[125] Akinleye A Chen Y Mukhi N Song Y Liu D. Ibrutinib and novel BTK inhibitors in clinical development. J Hematol Oncol. (2013) 6:59. 10.1186/1756-8722-6-59 23958373 PMC3751776

[126] Seiler T Hutter G Dreyling M. The emerging role of PI3K inhibitors in the treatment of hematological malignancies: preclinical data and clinical progress to date. Drugs. (2016) 76:639–46. 10.1007/s40265-016-0565-4 27052260

[127] Batlevi CL De Frank S Stewart C Hamlin PA Matasar MJ Gerecitano JF Phase I/II clinical trial of ibrutinib and buparlisib in relapsed/refractory diffuse large B-cell lymphoma, mantle cell lymphoma, and follicular lymphoma. J Clin Oncol. (2018) 36:7520 10.1200/JCO.2018.36.15_suppl.7520

[128] Assouline S Amrein L Banerji V Caplan S Owen CJ Hasegawa W A Phase II clinical trial of the pan PI3K inhibitor, buparlisib, for the treatment of relapsed and refractory chronic lymphocytic leukemia: canadian cancer trials group IND. 216. Blood. (2017) 130:4319.

[129] Younes A Salles G Martinelli G Bociek RG Barrigon DC Barca EG An open-label Phase II study of buparlisib (BKM120) in patients with relapsed and refractory Diffuse Large B-Cell Lymphoma (DLBCL), Mantle Cell Lymphoma (MCL) and Follicular Lymphoma (FL). Blood. (2015) 126.26160184

[130] Krause G Hassenruck F Hallek M. Copanlisib for treatment of B-cell malignancies: the development of a PI3K inhibitor with considerable differences to idelalisib. Drug Des Devel Ther. (2018) 12:2577–90. 10.2147/DDDT.S142406 30174412 PMC6109662

[131] Dreyling M Morschhauser F Bouabdallah K Bron D Cunningham D Assouline SE Phase II study of copanlisib, a PI3K inhibitor, in relapsed or refractory, indolent or aggressive lymphoma. Ann Oncol. (2017) 28:2169–78. 10.1093/annonc/mdx289 28633365 PMC5834070

[132] Dreyling M Santoro A Mollica L Leppa S Follows GA Lenz G Phosphatidylinositol 3-kinase inhibition by copanlisib in relapsed or refractory indolent lymphoma. J Clin Oncol. (2017) 35:3898–905. 10.1200/JCO.2017.75.4648 28976790

[133] Cheson BD O'Brien S Ewer MS Goncalves MD Farooki A Lenz G. Optimal management of adverse events from copanlisib in the treatment of patients with non-Hodgkin lymphomas. Clin Lymphoma Myeloma Leuk. (2019) 19:135–41. 10.1016/j.clml.2018.11.021 30584024 PMC6642803

[134] Dreyling M. A closer look at copanlisib. Clin Adv Hematol Oncol. (2018) 16:35–7. 29741503

[135] Gilbert JA. Idelalisib: targeting PI3Kdelta in B-cell malignancies. Lancet Oncol. (2014) 15:e108. 10.1016/S1470-2045(14)70052-X 24809089

[136] Miller BW Przepiorka D de Claro RA Lee K Nie L Simpson N. FDA approval: idelalisib monotherapy for the treatment of patients with follicular lymphoma and small lymphocytic lymphoma. Clin Cancer Res. (2015) 21:1525–9. 10.1158/1078-0432.CCR-14-2522 25645861

[137] Do B Mace M Rexwinkle A. Idelalisib for treatment of B-cell malignancies. Am J Health Syst Pharm. (2016) 73:547–55. 10.2146/ajhp150281 26933132

[138] Madanat YF Smith MR Almasan A Hill BT Idelalisib therapy of indolent B-cell malignancies: chronic lymphocytic leukemia and small lymphocytic or follicular lymphomas. Blood Lymphat Cancer. (2016) 6:1–6. 10.2147/BLCTT.S73530 27375364 PMC4929980

[139] Gopal AK Kahl BS de Vos S Wagner-Johnston ND Schuster SJ Jurczak WJ. PI3Kdelta inhibition by idelalisib in patients with relapsed indolent lymphoma. N Engl J Med. (2014) 370:1008–18. 10.1056/NEJMoa1314583 24450858 PMC4039496

[140] Gopal AK Fanale MA Moskowitz CH Shustov AR Mitra S Ye W. Phase II study of idelalisib, a selective inhibitor of PI3Kdelta, for relapsed/refractory classical Hodgkin lymphoma. Ann Oncol. (2017) 28:1057–63. 10.1093/annonc/mdx028 28327905 PMC6246229

[141] Gopal AK Kahl BS Flowers CR Martin P Ansell SM Abella-Dominicis E. Idelalisib is effective in patients with high-risk follicular lymphoma and early relapse after initial chemoimmunotherapy. Blood. (2017) 129:3037–9. 10.1182/blood-2016-12-757740 28325864

[142] Graf SA Gopal AK. Idelalisib for the treatment of non-Hodgkin lymphoma. Expert Opin Pharmacother. (2016) 17:265–74. 10.1517/14656566.2016.1135130 26818003 PMC4955805

[143] Salles G Schuster SJ de Vos S Wagner-Johnston ND Viardot A Blum KA. Efficacy and safety of idelalisib in patients with relapsed, rituximab- and alkylating agent-refractory follicular lymphoma: a subgroup analysis of a Phase 2 study. Haematologica. (2017) 102:e156–9. 10.3324/haematol.2016.151738 27979923 PMC5395130

[144] Williams A Baloga E Baran A Helber M Moore J Casulo C Increased risk of toxicity with novel PI3K delta inhibitor combinations compared with standard use in non-Hodgkin lymphoma patients. Blood. (2017) 130:4050.

[145] Bender MT Sanso LM Narula N Harvey BG Kaner RJ Pi3k delta inhibitor pulmonary toxicity. Am J Respir Crit Care Med. (2017) 195.

[146] Greenwell IB Ip A Cohen JB. PI3K inhibitors: understanding toxicity mechanisms and management. Oncology. (2017) 31:821–8. 29179250

[147] Liu D Mamorska-Dyga A. Syk inhibitors in clinical development for hematological malignancies. J Hematol Oncol. (2017) 10:145. 10.1186/s13045-017-0512-1 28754125 PMC5534090

[148] Coffey G Betz A DeGuzman F Pak Y Inagaki M Baker DC. The novel kinase inhibitor PRT062070 (Cerdulatinib) demonstrates efficacy in models of autoimmunity and B-cell cancer. J Pharmacol Exp Ther. (2014) 351:538–48. 10.1124/jpet.114.218164 25253883

[149] Ma J Xing W Coffey G Dresser K Lu K Guo A. Cerdulatinib, a novel dual SYK/JAK kinase inhibitor, has broad anti-tumor activity in both ABC and GCB types of diffuse large B cell lymphoma. Oncotarget. (2015) 6:43881–96. 10.18632/oncotarget.6316 26575169 PMC4791274

[150] Blunt MD Koehrer S Dobson RC Larrayoz M Wilmore S Hayman A. The dual Syk/JAK inhibitor cerdulatinib antagonizes B-cell receptor and microenvironmental signaling in chronic lymphocytic leukemia. Clin Cancer Res. (2017) 23:2313–24. 10.1158/1078-0432.CCR-16-1662 27697994 PMC5417366

[151] Guo A Lu P Coffey G Conley P Pandey A Wang YL Dual SYK/JAK inhibition overcomes ibrutinib resistance in chronic lymphocytic leukemia: cerdulatinib, but not ibrutinib, induces apoptosis of tumor cells protected by the microenvironment. Oncotarget. (2017) 8:12953–67. 10.18632/oncotarget.14588 28088788 PMC5355069

[152] Coffey G Rani A Betz A Pak Y Haberstock-Debic H Pandey A. PRT062607 achieves complete inhibition of the spleen tyrosine kinase at tolerated exposures following oral dosing in healthy volunteers. J Clin Pharmacol. (2017) 57:194–210. 10.1002/jcph.794 27406873 PMC5248591

[153] Flinn I Hamlin PA Strickland DK Pandey A Birrell M Coffey G Phase 1 open-label dose escalation study of the dual SYK/JAK inhibitor cerdulatinib (PRT062070) in patients with relapsed/refractory B-cell malignancies: safety profile and clinical activity. J Clin Oncol. (2015) 33:8531 10.1200/jco.2015.33.15_suppl.8531

[154] Hamlin PA Flinn I Wagner-Johnston N Burger JA Michelson G Pandey A Clinical and correlative results of a Phase 1 study of cerdulatinib (PRT062070) a dual SYK/JAK inhibitor in patients with relapsed/refractory B cell malignancies. Blood. (2015) 126.26160184

[155] Currie KS Kropf JE Lee T Blomgren P Xu J Zhao Z. Discovery of GS-9973, a selective and orally efficacious inhibitor of spleen tyrosine kinase. J Med Chem. (2014) 57:3856–73. 10.1021/jm500228a 24779514

[156] Sharman JP Kolibaba KS Shustov AR Giever TA Klein LM Nay D Results of a Phase 2 trial evaluating efficacy and safety of entospletinib (GS-9973) in patients with mantle cell lymphoma. Blood. (2016) 128:2963.

[157] Braselmann S Taylor V Zhao H Wang S Sylvain C Baluom M. R406, an orally available spleen tyrosine kinase inhibitor blocks fc receptor signaling and reduces immune complex-mediated inflammation. J Pharmacol Exp Ther. (2006) 319:998–1008. 10.1124/jpet.106.109058 16946104

[158] Flinn IW Bartlett NL Blum KA Ardeshna KM LaCasce AS Flowers CR A Phase II trial to evaluate the efficacy of fostamatinib in patients with relapsed or refractory diffuse large B-cell lymphoma (DLBCL). Eur J Cancer. (2016) 54:11–7. 10.1016/j.ejca.2015.10.005 26707592

[159] Friedberg JW Sharman J Sweetenham J Johnston PB Vose JM Lacasce A. Inhibition of Syk with fostamatinib disodium has significant clinical activity in non-Hodgkin lymphoma and chronic lymphocytic leukemia. Blood. (2010) 115:2578–85. 10.1182/blood-2009-08-236471 19965662 PMC2852362

[160] Herman SE Barr PM McAuley EM Liu D Wiestner A Friedberg JW. Fostamatinib inhibits B-cell receptor signaling, cellular activation and tumor proliferation in patients with relapsed and refractory chronic lymphocytic leukemia. Leukemia. (2013) 27:1769–73. 10.1038/leu.2013.37 23385377 PMC3920486

[161] Lam B Arikawa Y Cramlett J Dong Q de Jong R Feher V. Discovery of TAK-659 an orally available investigational inhibitor of Spleen Tyrosine Kinase (SYK). Bioorg Med Chem Lett. (2016) 26:5947–50. 10.1016/j.bmcl.2016.10.087 27839918

[162] Ciechanover A. The ubiquitin-proteasome proteolytic pathway. Cell. (1994) 79:13–21. 10.1016/0092-8674(94)90396-4 7923371

[163] Glickman MH Ciechanover A. The ubiquitin-proteasome proteolytic pathway: destruction for the sake of construction. Physiol Rev. (2002) 82:373–428. 10.1152/physrev.00027.2001 11917093

[164] Weathington NM Mallampalli RK. Emerging therapies targeting the ubiquitin proteasome system in cancer. J Clin Invest. (2014) 124:6–12. 10.1172/JCI71602 24382383 PMC3871250

[165] Ao N Chen Q Liu G. The small molecules targeting ubiquitin-proteasome system for cancer therapy. Comb Chem High Throughput Screen. (2017) 20:403–13. 10.2174/1386207320666170710124746 28699494

[166] Shen M Schmitt S Buac D Dou QP. Targeting the ubiquitin-proteasome system for cancer therapy. Expert Opin Ther Targets. (2013) 17:1091–108. 10.1517/14728222.2013.815728 23822887 PMC3773690

[167] Yang Y Kitagaki J Wang H Hou DX Perantoni AO. Targeting the ubiquitin-proteasome system for cancer therapy. Cancer Sci. (2009) 100:24–8. 10.1111/j.1349-7006.2008.01013.x 19037995 PMC2643214

[168] Soave CL Guerin T Liu J Dou QP. Targeting the ubiquitin-proteasome system for cancer treatment: discovering novel inhibitors from nature and drug repurposing. Cancer Metastasis Rev. (2017) 36:717–36. 10.1007/s10555-017-9705-x 29047025 PMC5722705

[169] Mikhael J Chang H Bortezomib: Proteasome inhibition as a novel mechanism of cancer therapy-implications for hematological malignancies. Lett Drug Des Discov. (2007) 4:82–6. 10.2174/157018007779422541

[170] Troch M Jonak C Mullauer L Puspok A Formanek M Hauff W. A Phase II study of bortezomib in patients with MALT lymphoma. Haematologica. (2009) 94:738–42. 10.3324/haematol.2008.001537 19336742 PMC2675689

[171] Lee EC Fitzgerald M Bannerman B Donelan J Bano K Terkelsen J. Antitumor activity of the investigational proteasome inhibitor MLN9708 in mouse models of B-cell and plasma cell malignancies. Clin Cancer Res. (2011) 17:7313–23. 10.1158/1078-0432.CCR-11-0636 21903769 PMC3443972

[172] Kupperman E Lee EC Cao Y Bannerman B Fitzgerald M Berger A. Evaluation of the proteasome inhibitor MLN9708 in preclinical models of human cancer. Cancer Res. (2010) 70:1970–80. 10.1158/0008-5472.CAN-09-2766 20160034

[173] Brayer J Baz R. The potential of ixazomib, a second-generation proteasome inhibitor, in the treatment of multiple myeloma. Ther Adv Hematol. (2017) 8:209–20. 10.1177/2040620717710171 28694935 PMC5495505

[174] Iannaccone A Bruno G Ravera A Gay F Salvini M Bringhen S. Evaluation of cardiovascular toxicity associated with treatments containing proteasome inhibitors in multiple myeloma therapy. High Blood Press Cardiovasc Prev. (2018) 25:209–18. 10.1007/s40292-018-0256-1 29582365

[175] Schmidt N Alloway RR Sadaka B Wall G Shields AR Woodle ES Intermediate-term global toxicity analysis of proteasome inhibitor-based antihumoral therapy. Am J Transpl. (2011) 11:192.

[176] Stansborough RL Gibson RJ. Proteasome inhibitor-induced gastrointestinal toxicity. Curr Opin Support Palliat Care. (2017) 11:133–7. 10.1097/SPC.0000000000000266 28333868

[177] Kuhn DJ Orlowski RZ Bjorklund CC. Second generation proteasome inhibitors: carfilzomib and immunoproteasome-specific inhibitors (IPSIs). Curr Cancer Drug Targets. (2011) 11:285–95. 10.2174/156800911794519725 21247387

[178] Dutcher JP. Mammalian target of rapamycin (mTOR) inhibitors. Curr Oncol Rep. (2004) 6:111–5. 10.1007/s11912-004-0022-5 14751088

[179] Rao RD Buckner JC Sarkaria JN. Mammalian target of rapamycin (mTOR) inhibitors as anti-cancer agents. Curr Cancer Drug Targets. (2004) 4:621–35. 10.2174/1568009043332718 15578919

[180] Smith SM. Clinical development of mTOR inhibitors: a focus on lymphoma. Rev Recent Clin Trials. (2007) 2:103–10. 10.2174/157488707780599366 18473994

[181] Dutton A Reynolds GM Dawson CW Young LS Murray PG. Constitutive activation of phosphatidyl-inositide 3 kinase contributes to the survival of Hodgkin's lymphoma cells through a mechanism involving Akt kinase and mTOR. J Pathol. (2005) 205:498–506. 10.1002/path.1725 15714459

[182] Hess G. mTOR inhibition in diffuse large B-cell lymphoma: new hope? Lancet Haematol. (2016) 3:e302–3. 10.1016/S2352-3026(16)30047-3 27374460

[183] Bennani NN LaPlant BR Ansell SM Habermann TM Inwards DJ Micallef IN Efficacy of the oral mTORC1 inhibitor everolimus in relapsed or refractory indolent lymphoma. Am J Hematol. (2017) 92:448–53. 10.1002/ajh.24671 28211162

[184] Haritunians T Mori A O'Kelly J Luong QT Giles FJ Koeffler HP. Antiproliferative activity of RAD001 (everolimus) as a single agent and combined with other agents in mantle cell lymphoma. Leukemia. (2007) 21:333–9. 10.1038/sj.leu.2404471 17136116

[185] Schoch LK Asiama A Zahurak M Shanbhag S Hurtt J Sawyer K. Pharmacokinetically-targeted dosed everolimus maintenance therapy in lymphoma patients. Cancer Chemother Pharmacol. (2018) 81:347–54. 10.1007/s00280-017-3499-y 29234922

[186] Johnston PB Pinter-Brown LC Warsi G White K Ramchandren R Phase 2 study of everolimus for relapsed or refractory classical Hodgkin lymphoma. Exp Hematol Oncol. (2018) 7:12 10.1186/s40164-018-0103-z 29774169 PMC5948762

[187] Johnston PB Inwards DJ Colgan JP Laplant BR Kabat BF Habermann TM. A Phase II trial of the oral mTOR inhibitor everolimus in relapsed Hodgkin lymphoma. Am J Hematol. (2010) 85:320–4. 10.1002/ajh.21664 20229590 PMC4420736

[188] Zent CS LaPlant BR Johnston PB Call TG Habermann TM Micallef IN. The treatment of recurrent/refractory chronic lymphocytic leukemia/small lymphocytic lymphoma (CLL) with everolimus results in clinical responses and mobilization of CLL cells into the circulation. Cancer. (2010) 116:2201–7. 10.1002/cncr.25005 20166206 PMC2861142

[189] Xu ZZ Wang WF Fu WB Wang AH Liu ZY Chen LY. Combination of rituximab and mammalian target of rapamycin inhibitor everolimus (RAD001) in diffuse large B-cell lymphoma. Leuk Lymphoma. (2014) 55:1151–7. 10.3109/10428194.2013.823492 23841505

[190] Barnes JA Jacobsen E Feng Y Freedman A Hochberg EP LaCasce AS. Everolimus in combination with rituximab induces complete responses in heavily pretreated diffuse large B-cell lymphoma. Haematologica. (2013) 98:615–9. 10.3324/haematol.2012.075184 23144193 PMC3659994

[191] Kwitkowski VE Prowell TM Ibrahim A Farrell AT Justice R Mitchell SS. FDA approval summary: temsirolimus as treatment for advanced renal cell carcinoma. Oncologist. (2010) 15:428–35. 10.1634/theoncologist.2009-0178 20332142 PMC3227966

[192] Grossheim U Temsirolimus (Torisel (R)): first approved drug for the advanced mantle cell lymphoma. Onkologie. (2010) 33:275.

[193] Witzens-Harig M Memmer ML Dreyling M Hess G. A Phase I/II trial to evaluate the safety, feasibility and activity of salvage therapy consisting of the mTOR inhibitor temsirolimus added to standard therapy of rituximab and DHAP for the treatment of patients with relapsed or refractory diffuse large cell B-Cell lymphoma - the STORM trial. BMC Cancer. (2013) 13:308. 10.1186/1471-2407-13-308 23799873 PMC3701613

[194] Witzens-Harig M Viardot A Keller U Buske C Crombe A Hoenig E Safety and clinical activity of temsirolimus in combination with rituximab and DHAP in patients with relapsed or refractory diffuse large B-cell lymphoma - report of the prospective, multicenter Phase II STORM trial. Blood. (2016) 128:3028 10.1002/hon.2438_52

[195] Sofroniadou S Goldsmith D. Mammalian target of rapamycin (mTOR) inhibitors: potential uses and a review of haematological adverse effects. Drug Saf . (2011) 34:97–115. 10.2165/11585040-000000000-00000 21247219

[196] Pallet N Legendre C. Adverse events associated with mTOR inhibitors. Expert Opin Drug Saf . (2013) 12:177–86. 10.1517/14740338.2013.752814 23252795

[197] Kaplan B Qazi Y Wellen JR. Strategies for the management of adverse events associated with mTOR inhibitors. Transplant Rev. (2014) 28:126–33. 10.1016/j.trre.2014.03.002 24685370

[198] Aster JC Longtine JA. Detection of BCL2 rearrangements in follicular lymphoma. Am J Pathol. (2002) 160:759–63. 10.1016/S0002-9440(10)64897-3 11891173 PMC1867166

[199] Weisenburger DD Gascoyne RD Bierman PJ Shenkier T Horsman DE Lynch JC. Clinical significance of the t(14;18) and BCL2 overexpression in follicular large cell lymphoma. Leuk Lymphoma. (2000) 36:513–23. 10.3109/10428190009148399 10784396

[200] Monni O Franssila K Joensuu H Knuutila S. BCL2 overexpression in diffuse large B-cell lymphoma. Leuk Lymphoma. (1999) 34:45–52. 10.3109/10428199909083379 10350331

[201] Bcl2 (B cell lymphoma) Bull Du Cancer. (1998) 85:738. 9770595

[202] Harris MM Coon Z Alqaeisoom N Swords B Holub JM. Targeting anti-apoptotic Bcl2 proteins with scyllatoxin-based BH3 domain mimetics. Org Biomol Chem. (2016) 14:440–6. 10.1039/C5OB02080H 26563651

[203] Roberts AW Huang D. Targeting BCL2 with BH3 mimetics: basic science and clinical application of venetoclax in chronic lymphocytic leukemia and related B cell malignancies. Clin Pharmacol Ther. (2017) 101:89–98. 10.1002/cpt.553 27806433 PMC5657403

[204] Adams CM Clark-Garvey S Porcu P Eischen CM. Targeting the Bcl-2 family in B cell lymphoma. Front Oncol. (2018) 8:636. 10.3389/fonc.2018.00636 30671383 PMC6331425

[205] Paoluzzi L Gonen M Gardner JR Mastrella J Yang D Holmlund J. Targeting Bcl-2 family members with the BH3 mimetic AT-101 markedly enhances the therapeutic effects of chemotherapeutic agents in *in vitro* and *in vivo* models of B-cell lymphoma. Blood. (2008) 111:5350–8. 10.1182/blood-2007-12-129833 18292288

[206] Cang S Iragavarapu C Savooji J Song Y Liu D. ABT-199 (venetoclax) and BCL-2 inhibitors in clinical development. J Hematol Oncol. (2015) 8:129. 10.1186/s13045-015-0224-3 26589495 PMC4654800

[207] Stolz C Hess G Hahnel PS Grabellus F Hoffarth S Schmid KW. Targeting Bcl-2 family proteins modulates the sensitivity of B-cell lymphoma to rituximab-induced apoptosis. Blood. (2008) 112:3312–21. 10.1182/blood-2007-11-124487 18689543

[208] Pham LV Huang S Zhang H Zhang J Bell T Zhou S. Strategic therapeutic targeting to overcome venetoclax resistance in aggressive B-cell lymphomas. Clin Cancer Res. (2018) 24:3967–80. 10.1158/1078-0432.CCR-17-3004 29666304

[209] Roberts AW Davids MS Pagel JM Kahl BS Puvvada SD Gerecitano JF. Targeting BCL2 with venetoclax in relapsed chronic lymphocytic leukemia. N Engl J Med. (2016) 374:311–22. 10.1056/NEJMoa1513257 26639348 PMC7107002

[210] Seymour JF Kipps TJ Eichhorst B Hillmen P D'Rozario J Assouline S Venetoclax-rituximab in relapsed or refractory chronic lymphocytic leukemia. N Engl J Med. (2018) 378:1107–20. 10.1056/NEJMoa1713976 29562156

[211] Seymour JF Mobasher M Kater AP Venetoclax-rituximab in chronic lymphocytic leukemia. N Engl J Med. (2018) 378:2143–4. 10.1056/NEJMc1805135 29847765

[212] Kater AP Seymour JF Hillmen P Eichhorst B Langerak AW Owen C. Fixed duration of venetoclax-rituximab in relapsed/refractory chronic lymphocytic leukemia eradicates minimal residual disease and prolongs survival: post-treatment follow-up of the MURANO Phase III study. J Clin Oncol. (2019) 37:269–77. 10.1200/JCO.18.01580 30523712

[213] Seymour JF Kipps TJ Eichhorst B Hillmen P D'Rozario J Assouline S MURANO trial establishes feasibility of time-limited Venetoclax-Rituximab (VenR) combination therapy in Relapsed/Refractory (R/R) Chronic Lymphocytic Leukemia (CLL). Blood. (2018) 132:184 10.1182/blood-2018-184

[214] Mato AR Tam CS Allan JN Brander DM Pagel JM Ujjani CS Disease and patient characteristics, patterns of care, toxicities, and outcomes of Chronic Lymphocytic Leukemia (CLL) patients treated with venetoclax: a multicenter study of 204 patients. Blood. (2017) 130:4315.

[215] Davids MS Gerecitano J Potluri J Cerri E Kim SY Steinberg M Integrated safety analysis of venetoclax monotherapy in Chronic Lymphocytic Leukemia (CLL). Haematologica. (2016) 101:59.

[216] Brander DM Choi MY Roberts AW Ma S Lash LL Verdugo M Detailed safety analysis of venetoclax combined with rituximab in patients with relapsed/refractory chronic lymphocytic leukemia. Blood. (2016) 128:2033.27609643

[217] Evans L. The American Society of Hematology−46th Annual Meeting and Exposition. HDAC, Flt and farnesyl transferase inhibitors. IDrugs. (2005) 8:4–6. 15650931

[218] Prince HM Bishton MJ Harrison SJ. Clinical studies of histone deacetylase inhibitors. Clin Cancer Res. (2009) 15:3958–69. 10.1158/1078-0432.CCR-08-2785 19509172

[219] Mottamal M Zheng S Huang TL Wang G. Histone deacetylase inhibitors in clinical studies as templates for new anticancer agents. Molecules. (2015) 20:3898–941. 10.3390/molecules20033898 25738536 PMC4372801

[220] Younes A Berdeja JG Patel MR Flinn I Gerecitano JF Neelapu SS Safety, tolerability, and preliminary activity of CUDC-907, a first-in-class, oral, dual inhibitor of HDAC and PI3K, in patients with relapsed or refractory lymphoma or multiple myeloma: an open-label, dose-escalation, Phase 1 trial. Lancet Oncol. (2016) 17:622–31. 10.1016/S1470-2045(15)00584-7 27049457 PMC5494693

[221] Younes A Berdeja J Patel M Kelly K Flinn I Gerecitano J. Phase 1 trial of Cudc-907, a novel, oral dual inhibitor of Hdac and Pi3k: updated assessment of patients with relapsed or refractory diffuse large B-cell lymphoma, including double expressor lymphoma. Haematologica. (2016) 101:175. 29722657

[222] Younes A Flinn IW Oki Y Copland A Fattaey A Lai CJ A first-in-man Phase 1 study Of CUDC-907, a first-in-class chemically-designed dual inhibitor Of PI3K and HDAC in patients with refractory or relapsed lymphoma and multiple myeloma. Blood. (2013) 122:4363–5.

[223] Younes A Oki Y Bociek RG Kuruvilla J Fanale M Neelapu S. Mocetinostat for relapsed classical Hodgkin's lymphoma: an open-label, single-arm, Phase 2 trial. Lancet Oncol. (2011) 12:1222–8. 10.1016/S1470-2045(11)70265-0 22033282 PMC5042214

[224] Boumber Y Younes A Garcia-Manero G. Mocetinostat (MGCD0103): a review of an isotype-specific histone deacetylase inhibitor. Expert Opin Investig Drugs. (2011) 20:823–9. 10.1517/13543784.2011.577737 21554162 PMC5206967

[225] Batlevi CL Crump M Andreadis C Rizzieri D Assouline SE Fox S A Phase 2 study of mocetinostat, a histone deacetylase inhibitor, in relapsed or refractory lymphoma. Br J Haematol. (2017) 178:434–41. 10.1111/bjh.14698 28440559 PMC5576135

[226] Straus DJ Hamlin PA Matasar MJ Lia Palomba M Drullinsky PR Zelenetz AD Phase I/II trial of vorinostat with rituximab, cyclophosphamide, etoposide and prednisone as palliative treatment for elderly patients with relapsed or refractory diffuse large B-cell lymphoma not eligible for autologous stem cell transplantation. Br J Haematol. (2015) 168:663–70. 10.1111/bjh.13195 25316653

[227] Ogura M Ando K Suzuki T Ishizawa K Oh SY Itoh K A multicentre Phase II study of vorinostat in patients with relapsed or refractory indolent B-cell non-Hodgkin lymphoma and mantle cell lymphoma. Br J Haematol. (2014) 165:768–76. 10.1111/bjh.12819 24617454 PMC4282031

[228] Seo YH. Dual inhibitors against topoisomerases and histone deacetylases. J Cancer Prev. (2015) 20:85–91. 10.15430/JCP.2015.20.2.85 26151040 PMC4492363

[229] Subramanian S Bates SE Wright JJ Espinoza-Delgado I Piekarz RL. Clinical toxicities of histone deacetylase inhibitors. Pharmaceuticals. (2010) 3:2751–67. 10.3390/ph3092751 27713375 PMC4034096

[230] Nastoupil LJ Lunning MA Vose JM Schreeder MT Siddiqi T Flowers CR. Tolerability and activity of ublituximab, umbralisib, and ibrutinib in patients with chronic lymphocytic leukaemia and non-Hodgkin lymphoma: a Phase 1 dose escalation and expansion trial. Lancet Haematol. (2019) 6:e100–9. 10.1016/S2352-3026(18)30216-3 30709431 PMC11827449

[231] Younes A Brody J Carpio C Lopez-Guillermo A Ben-Yehuda D Ferhanoglu B. Safety and activity of ibrutinib in combination with nivolumab in patients with relapsed non-Hodgkin lymphoma or chronic lymphocytic leukaemia: a Phase 1/2a study. Lancet Haematol. (2019) 6:e67–78. 10.1016/S2352-3026(18)30217-5 30642819

[232] Ganjoo KN de Vos S Pohlman BL Flinn IW Forero-Torres A Enas NH. Phase 1/2 study of ocaratuzumab, an Fc-engineered humanized anti-CD20 monoclonal antibody, in low-affinity FcgammaRIIIa patients with previously treated follicular lymphoma. Leuk Lymphoma. (2015) 56:42–8. 10.3109/10428194.2014.911859 24717109

[233] Czuczman MS Fayad L Delwail V Cartron G Jacobsen E Kuliczkowski K. Ofatumumab monotherapy in rituximab-refractory follicular lymphoma: results from a multicenter study. Blood. (2012) 119:3698–704. 10.1182/blood-2011-09-378323 22389254

[234] Westin JR Chu F Zhang M Fayad LE Kwak LW Fowler N. Safety and activity of PD1 blockade by pidilizumab in combination with rituximab in patients with relapsed follicular lymphoma: a single group, open-label, Phase 2 trial. Lancet Oncol. (2014) 15:69–77. 10.1016/S1470-2045(13)70551-5 24332512 PMC3922714

[235] Bartlett NL Costello BA LaPlant BR Ansell SM Kuruvilla JG Reeder CB. Single-agent ibrutinib in relapsed or refractory follicular lymphoma: a Phase 2 consortium trial. Blood. (2018) 131:182–90. 10.1182/blood-2017-09-804641 29074501 PMC5757691

[236] Davids MS Roberts AW Seymour JF Pagel JM Kahl BS Wierda WG Phase I first-in-human study of venetoclax in patients with relapsed or refractory non-Hodgkin lymphoma. J Clin Oncol. (2017) 35:826–33. 10.1200/JCO.2016.70.4320 28095146 PMC5455685

[237] Noy A de Vos S Thieblemont C Martin P Flowers CR Morschhauser F. Targeting Bruton tyrosine kinase with ibrutinib in relapsed/refractory marginal zone lymphoma. Blood. (2017) 129:2224–32. 10.1182/blood-2016-10-747345 28167659 PMC5399483

[238] Ansell SM Hurvitz SA Koenig PA LaPlant BR Kabat BF Fernando D. Phase I study of ipilimumab, an anti-CTLA-4 monoclonal antibody, in patients with relapsed and refractory B-cell non-Hodgkin lymphoma. Clin Cancer Res. (2009) 15:6446–53. 10.1158/1078-0432.CCR-09-1339 19808874 PMC2763019

[239] Armand P Nagler A Weller EA Devine SM Avigan DE Chen YB. Disabling immune tolerance by programmed death-1 blockade with pidilizumab after autologous hematopoietic stem-cell transplantation for diffuse large B-cell lymphoma: results of an international Phase II trial. J Clin Oncol. (2013) 31:4199–206. 10.1200/JCO.2012.48.3685 24127452 PMC4878008

[240] Wang ML Lee H Chuang H Wagner-Bartak N Hagemeister F Westin J. Ibrutinib in combination with rituximab in relapsed or refractory mantle cell lymphoma: a single-centre, open-label, Phase 2 trial. Lancet Oncol. (2016) 17:48–56. 10.1016/S1470-2045(15)00438-6 26640039

[241] Younes A Salles G Martinelli G Bociek RG Barrigon DC Barca EG Pan-phosphatidylinositol 3-kinase inhibition with buparlisib in patients with relapsed or refractory non-Hodgkin lymphoma. Haematologica. (2017) 102:2104–12. 10.3324/haematol.2017.169656 28971900 PMC5709110

[242] Oki Y Kelly KR Flinn I Patel MR Gharavi R Ma A. CUDC-907 in relapsed/refractory diffuse large B-cell lymphoma, including patients with MYC-alterations: results from an expanded Phase I trial. Haematologica. (2017) 102:1923–30. 10.3324/haematol.2017.172882 28860342 PMC5664396

[243] Wang M Schuster SJ Phillips T Lossos IS Goy A Rule S. Observational study of lenalidomide in patients with mantle cell lymphoma who relapsed/progressed after or were refractory/intolerant to ibrutinib (MCL-004). J Hematol Oncol. (2017) 10:171. 10.1186/s13045-017-0537-5 29096668 PMC5668956

[244] Jerkeman M Eskelund CW Hutchings M Raty R Wader KF Laurell A. Ibrutinib, lenalidomide, and rituximab in relapsed or refractory mantle cell lymphoma (PHILEMON): a multicentre, open-label, single-arm, Phase 2 trial. Lancet Haematol. (2018) 5:e109–16. 10.1016/S2352-3026(18)30018-8 29396091

[245] Zhang H Chen J. Current status and future directions of cancer immunotherapy. J Cancer. (2018) 9:1773–81. 10.7150/jca.24577 29805703 PMC5968765

